# Adolescent circadian rhythm disruption increases reward and risk-taking

**DOI:** 10.3389/fnins.2024.1478508

**Published:** 2024-12-16

**Authors:** Lauren M. DePoy, Chelsea A. Vadnie, Kaitlyn A. Petersen, Madeline R. Scott, Wei Zong, RuoFei Yin, Ross C. Matthaei, Fernanda Juarez Anaya, Callie I. Kampe, George C. Tseng, Colleen A. McClung

**Affiliations:** ^1^Department of Psychiatry, Translational Neuroscience Program, University of Pittsburgh School of Medicine, Pittsburgh, PA, United States; ^2^Center for Neuroscience, University of Pittsburgh, Pittsburgh, PA, United States; ^3^David O. Robbins Neuroscience Program, Department of Psychology, Ohio Wesleyan University, Delaware, OH, United States; ^4^Department of Biostatistics, University of Pittsburgh, Pittsburgh, PA, United States

**Keywords:** circadian disruption, adolescence, cocaine, anxiety, risk-taking, reward

## Abstract

**Introduction:**

Circadian rhythm disturbances have long been associated with the development of psychiatric disorders, including mood and substance use disorders. Adolescence is a particularly vulnerable time for the onset of psychiatric disorders and for circadian rhythm and sleep disruptions. Preclinical studies have found that circadian rhythm disruption (CRD) impacts the brain and behavior, but this research is largely focused on adult disruptions. Here, we hypothesized that adolescent CRD would have a greater effect on psychiatric-related behaviors, relative to adult disruption.

**Methods:**

We determined the long-term behavioral and neurobiological effects of CRD during early adolescence by exposing mice to 12 h shifts in the light/dark cycle. Adult mice were exposed to the same CRD paradigm. Behavior testing began approximately 4 weeks later for both groups. To identify possible mechanisms, we also measured gene expression in brain regions relevant to circadian rhythms, mood and reward.

**Results:**

CRD during early adolescence, but not adulthood, persistently increased exploratory drive (risk-taking behavior) and cocaine preference when tested later in life. Interestingly, we found sex differences when intravenous cocaine self-administration was tested. While female mice with a history of adolescent CRD had a greater propensity to self-administer cocaine, as well as increased motivation and cue-induced reinstatement, male adolescent CRD mice had reduced motivation and extinction responding. Importantly, we found that transcripts in the SCN were affected by adolescent CRD and these were largely distinct across sex.

**Conclusion:**

Overall, adolescent CRD in mice caused persistent increases in risky behavior, cocaine reward and cocaine self-administration, which suggests that CRD during adolescence may predispose individuals toward substance use disorders. Future research is required to elucidate how adolescent CRD affects behaviors relevant to mood-and substance use-related disorders across the 24-h day, as well as to identify intervention strategies to alleviate disruption during adolescence and novel therapeutic approaches once symptoms have begun.

## 1 Introduction

Psychiatric disorders and circadian rhythm disruptions (CRD) commonly co-occur. Variations and disruptions of rhythms (e.g., evening chronotypes, misaligned rhythms, and delayed rhythms) are frequently described in psychiatric disorders (Souêtre et al., 1989; McClung, 2007; Emens et al., 2009; Merikanto et al., 2015; Vadnie and McClung, 2017), including substance use disorders (SUDs; Logan et al., 2014; DePoy et al., 2017). On the other hand, those with disrupted sleep or circadian rhythms commonly exhibit increased symptoms of psychiatric disorders, such as increased risk-taking and drug use (Wittmann et al., 2006; Gowen et al., 2019; Taylor et al., 2020; Pasch et al., 2012; Hasler et al., 2017; Hasler et al., 2016), suggesting a bidirectional relationship between psychiatric disorders and CRD. Supporting this, environmental (e.g., light at night) and genetic insults to circadian rhythms are linked to altered mood (Benedetti et al., 2003, 2008; Easton et al., 2003; Johansson et al., 2003; Roybal et al., 2007; Mansour et al., 2009; Lavebratt et al., 2010) and substance use (Dong et al., 2011; Forbes et al., 2012; Ozburn et al., 2012, 2013; Blomeyer et al., 2013; Gamsby et al., 2013; Bi et al., 2014; Baranger et al., 2016) across species. External or internal disruptions can perturb circadian rhythms by impacting the core molecular clock, a series of interlocking transcriptional-translational feedback loops found in almost every cell in the body (Reppert and Weaver, 2002; Ko and Takahashi, 2006; Takahashi, 2017), which controls physiological processes and behavior across the light/dark (LD) cycle. The molecular clock is largely regulated by the CLOCK protein, which dimerizes with ARNTL (BMAL1) to control transcription of many genes including the Period (*Per*) and Cryptochrome (*Cry*) genes. After translation, the PER and CRY proteins enter the nucleus and inhibit the transcriptional activity of CLOCK/BMAL1, closing the negative feedback loop after around 24 hours (h; Ko and Takahashi, 2006).

Additionally, the suprachiasmatic nucleus (SCN), a brain region located in the hypothalamus, is known as the principal pacemaker of the brain and is responsible for the generation and maintenance of circadian rhythms across the brain and periphery. Not only can circadian disruption cause structural and functional changes to the SCN (Iglesia et al., 2004; Yan, 2011), but notably, preclinical studies support a role for the SCN in regulating anxiety-like and mood-related behaviors (Landgraf et al., 2016; Boehler et al., 2021; Whylings et al., 2021; Vadnie et al., 2022). Other regions likely to be involved in the effects of CRD on behaviors associated with psychiatric disorders include the prefrontal cortex (PFC) and nucleus accumbens (NAc). In animal models, circadian disruptions via clock gene knockouts and knockdowns have been shown to disrupt dopamine signaling rich areas, such as the NAc, and alter a variety of mood- and reward-related behaviors (Andretic et al., 1999; Falcón and McClung, 2009; Ozburn et al., 2012, 2017; Logan et al., 2014; Parekh and McClung, 2016; Parekh et al., 2019; DePoy et al., 2020). Further, in the PFC, a major site of executive function and cognitive control over behavior, neuronal activity and circadian gene expression are altered following exposure to a disrupted LD cycle (Karatsoreos et al., 2011; Otsuka et al., 2020; Roberts and Karatsoreos, 2023).

Preclinical studies have focused on investigating the effects of disturbing rhythms during adulthood or before weaning, but not during adolescence (Karatsoreos, Bhagat et al., 2011; LeGates et al., 2012; McGowan and Coogan, 2013; Borniger et al., 2014; Ben-Hamo et al., 2016; Chen et al., 2021b; Ameen et al, 2022). However, adolescence is a particularly vulnerable time for both psychiatric disorders and circadian disruptions. As reward-related brain regions mature faster than areas important for top-down cognitive control (Mills et al., 2014) and individuals seek independence from parents and more social interaction with peers (Steinberg and Silverberg, 1986), adolescence is typically a time of increased risk-taking and novelty-seeking behavior (Spear, 2000). Adolescents are also vulnerable to circadian disruptions due to early morning school, work and societal obligations conflicting with their preferred delayed sleep and wake times, resulting in misaligned rhythms and reductions in sleep which have been shown to negatively impact mental and physical health (Wahistrom, 2002; Owens et al., 2010; Touitou, 2013; Boergers et al., 2014; Borisenkov et al., 2022). Notably, circadian rhythms are delayed in adolescents across different countries and cultures (Thorleifsdottir et al., 2002; Roenneberg et al., 2004; Yang et al., 2005; Crowley et al., 2014) and phase-shifts are also found in other species, including rodents (Golub et al., 1996; Hummer et al., 2007; Hagenauer et al., 2011), suggesting this delay is biologically driven.

In rodents, adolescence is most broadly defined as the period from weaning postnatal day 21, (P21) to sexual maturity (P60), with most adolescent-typical neurobehavioral characteristics being present from P28-42 (Spear, 2000). During adolescence, there are significant changes occurring with neural circuitry and neurotransmitter systems. A few recent studies suggest that CRD during adolescence can induce lasting behavioral and neurobiological effects, indicating there is a need to further study the impact of circadian disruption during this developmental period when many psychiatric disorders start to arise (Chen et al., 2021a; Cloutier et al., 2022; Gonzalez et al., 2023; Bonilla et al., 2024; Jameson et al., 2024). Investigating the long-term impact of circadian rhythm disruption on behavior and the neurobiology of relevant brain regions may help us understand how psychiatric disorders develop and how they can be better prevented and treated.

The bidirectional relationship between psychiatric disorders and CRD begins during adolescence, a vulnerable time for engaging in risky behavior, such as initiating substance use, and suffering from CRD. In this study, we sought to determine the long-term behavioral and neurobiological effects of CRD during early adolescence and adulthood by exposing mice to 12 h shifts in the LD cycle. This type of light cycle paradigm is known to reduce exploratory drive and novelty seeking in adult mice (McGowan and Coogan, 2013) as well as shift and dampen the amplitude of rhythms (Kim et al., 2018). These types of rhythm disturbances have been implicated in psychiatric disorders in humans (Souêtre et al., 1989; Lewy, 2010; Hasler et al., 2017). We hypothesized that adolescent CRD would have a more profound effect on psychiatric-related behaviors relative to adult disruption. To identify possible mechanisms by which circadian disruption during adolescence affects behaviors later in life, we also measured gene expression in brain regions relevant to circadian rhythms, mood and reward, the SCN, PFC and NAc, respectively.

## 2 Materials and methods

### 2.1 Animals and housing conditions

Male and female C57BL/6 J mice were obtained from The Jackson Laboratory (000664) and then bred in house. Mice were maintained on a 12:12 light/dark (LD) schedule with lights on [Zeitgeber Time (ZT0)] at 0700 except for those exposed to CRD (described below). Behavioral testing occurred during the light phase from ZT2-10. Food and water were provided *ad libitum* unless otherwise indicated. Procedures were approved by the University of Pittsburgh Institutional Animal Care and Use Committee.

### 2.2 Circadian rhythm disruption

Adolescent CRD began at P28 ± 1, when light cycles were shifted 12 h to a 12:12 schedule with lights on at 1900. After 3 days, the light cycle was shifted back by 12 h to a 12:12 schedule with lights on at 0700. The 12 h shifts of the light cycle continued every 3 days, resulting in 4 complete reversals of the LD cycle spanning P28-37. Following adolescent CRD, mice were returned to a fixed LD schedule (ZT0 = 0700) and behavioral testing began approximately 4 weeks later, in adulthood. Due to the nature of this shifting paradigm, each experimental group was housed together, and mice were pseudo-randomized to each behavior testing group.

Adult mice experienced CRD as described above from P62-71. Following CRD, mice were returned to a fixed LD schedule (ZT0 = 0700), and behavioral testing began approximately 4 weeks later. Control adolescent and adult mice were physically disturbed at the same ages and times of day with matched cage handling but received no change to their LD schedule.

### 2.3 Drugs

Cocaine hydrochloride was provided by the National Institute on Drug Abuse. Animals were injected with 2.5, 5 or 10 mg/kg (intraperitoneal, i.p.; volume 10 mL/kg) for conditioned place preference testing and 0–1 mg/kg/infusion for cocaine self-administration.

### 2.4 Behavioral testing

Mice used for behavior testing were subjected to only 1 of the 3 different groups of behavior testing.

*Group 1* mice were used in a battery of tests in the following order to assess locomotor activity, exploratory drive [open field, elevated plus maze (EPM), light/dark (LD) box], spatial working memory (T-maze) and helplessness-like behavior (forced swim test, FST). Behavior testing took place every other day.

*Group 2* mice were tested for reward-related behavior. These mice were tested for sucrose preference and cocaine conditioned place preference (CPP).

*Group 3* mice were assessed for operant food self-administration and intravenous cocaine self-administration.

#### 2.4.1 Locomotor response to novelty

Mice were placed in clear boxes (field dimensions: 9.5″ x 18.0″) equipped with photobeams (Kinder Scientific Smart Cage Rack System, Poway, CA). Total horizontal distance traveled over 120 minutes (min) was measured for each mouse under standard room lighting.

#### 2.4.2 Open field

Mice were placed in the corner of a large plastic arena (52 × 52 × 25 cm) with black walls and a clear bottom. The center was a 24 cm x 24 cm square in the middle of the box that was designated with taped gridlines. Mice were allowed to freely explore the box for 10 min. Behavior was video recorded under dim white light (~20 lux). Center entries and center time were quantified using Ethovision XT (Noldus, Leesburg, VA, United States).

#### 2.4.3 Elevated plus maze

As previously described (Vadnie et al., 2022), mice were placed in the center of the maze facing an open arm in dim light (~20 lux). The EPM consisted of 2 plastic open arms perpendicular to two closed arms (30 × 5 cm) and was elevated above the ground (81 cm). Behavior was recorded for 10 min and video tracking software (Ethovision XT; Noldus, Leesburg, VA, United States) was used to quantify the time spent in the open arms, as well as the number of entries into each arm.

#### 2.4.4 Light/dark box

Clear boxes (Kinder Scientific Smart Cage Rack System; field dimensions: 9.5″ x 18.0″) were divided into two equally sized chambers, a black opaque chamber kept in the dark with a lid and a brightly lit chamber (~880 lux). An opening was present between the two chambers that was equipped with a door. Mice were first placed in the dark chamber for 2 min and then the door was opened between the two sides. Mice were allowed to explore both chambers for 20 min. Photobeams were used to measure the number of entries into and time spent in the light side.

#### 2.4.5 T-maze

Testing in the T-maze took place under dim white light (~40 lux). The assay consisted of 5 trials made up of 2 runs (a sequestered and a non-sequestered run). For both runs the mouse was placed in the starting arm of the T-maze facing toward the wall, away from the two choice arms. For the first sequestered run of the trial, the mouse was given 2 min to select an arm. Once the mouse had all four paws in an arm, the door was closed so that the mouse was sequestered in the arm for 30 seconds (s). The mouse was then removed from the maze and placed in a clean holding cage for 20 s. Any urine or feces were removed from the T-maze to reduce scent cues. For the non-sequestered run, the mouse was again placed back in the starting arm facing toward the wall, away from the choice arms. The mouse was given 2 min to select an arm. Once the mouse selected an arm and had all 4 paws in the arm, it was placed back in the holding cage for 20 s. A spontaneous alternation was considered when the mouse selected the arm opposite to the one it was sequestered in. % spontaneous alternations were calculated as the number of alternating trials divided by the total completed trials multiplied by 100. If the mouse failed to select an arm, the mouse was placed back in the holding cage for 1 min. Then the mouse was permitted to re-do the trial. If the mouse still failed to select an arm, it was returned to a holding cage for 1 min and then was moved on to the next trial. Testing ended after 5 trials or after the mouse failed to select an arm for 3 consecutive runs. No mouse failed the test completely.

#### 2.4.6 Forced swim test

Mice were individually placed into glass beakers of water (25°C; 18 cm depth) that were separated by dividers to prevent the mice from observing each other during testing. Behavior was video recorded for 6 min. Time spent struggling during the last 4 min of the test and latency to immobility were scored by a blinded, trained observer. Struggling was defined as any movement that was not for the sole purpose of keeping the mouse afloat. Immobility time was calculated by subtracting the time spent struggling from the total time (4 min).

#### 2.4.7 Conditioned place preference

As previously described (Ozburn et al., 2015) mice were first habituated to a testing room for 30 min. On day 1, a pre-conditioning test was conducted, wherein mice were placed in the center of a 3-chamber box. The outer two chambers were distinct with visual and tactile differences. Time in each chamber was recorded over the 20-min session. On the subsequent 4 days, mice were injected with either saline or cocaine and restricted to one of the outer chambers. Saline was injected on days 2 and 4, and cocaine was injected on days 3 and 5 (2.5, 5 or 10 mg/kg, 10 mL/kg, NIDA drug consortium). A biased design was used, since >50% of mice showed a chamber bias during the pre-test, wherein the preferred chamber (>10% preference) was paired with saline or chambers were assigned pseudo-randomly if no side preference was found.

#### 2.4.8 Sucrose preference

Mice were habituated to two bottles containing water or a sucrose solution (0.5% sucrose w/v). After 24 h, bottle position was switched to minimize side preferences. After another 24 h, mice were individually housed and exposed to water and sucrose bottles. Mice were undisturbed overnight, for 18 h, and bottles were weighed to determine consumption of sucrose and water. Control cages were sham handled and fluid loss was recorded. Percent preference for sucrose consumption was calculated with 50% indicating no preference.

#### 2.4.9 Intravenous cocaine self-administration

Mice were first restricted to 85% of their free-feeding weight for food self-administration. Mice were trained to respond for chocolate flavored food pellets (20 mg, grain-based precision pellets, Bio-Serv) in Med-Associates operant conditioning chambers. Responding on one lever was reinforced using a fixed ratio 1 (FR1) schedule. A cue light was illuminated over the active lever for the duration of the experiment. Responses on the inactive lever had no programmed consequences but were recorded. Sessions ended at 60 min or when a maximum of 30 pellets were acquired. Mice were trained for at least five sessions or until they acquired ≥25 pellets for three consecutive sessions.

Mice then underwent jugular catheterization. Mice were anesthetized with isoflurane. As previously described (DePoy et al., 2020), the dorsal and ventral sides were shaved and disinfected. The right jugular vein was exposed by blunt dissection and a sterile polyurethane catheter was placed and secured to the vein. The catheter was exteriorized posterior to the scapulae via a felt mesh mount (Instech). The dorsal and ventral wounds were sutured, and mice were pair housed for the duration of the experiment, unless fighting or uneven sample sizes necessitated single housing. Mice received analgesic treatment (Rimadyl 5 mg/kg) following surgery and post-operatively for 2 days. Mice recover for 6–7 days (d) before intravenous self-administration training begins. Catheters were maintained by infusing catheters daily with 0.05 mL gentamicin (0.33 mg/mL) and heparinized saline (30 USP/ml) containing baytril (0.5 mg/kg). Catheter patency was tested approximately once per week using 0.05 mL brevital (3 mg/mL), mice that failed to lose muscle tone were excluded.

After recovery from surgery, mice were trained to respond on an FR1 schedule for cocaine (0.5 mg/kg/infusion, 30 μL over 1.7 s) on the previously inactive lever from food training (Ozburn et al., 2012). Cocaine was delivered through an armored tether connected to a swivel and syringe pump. Mice were tested 6 d/week with the last day being reserved for patency testing. Drug delivery culminated in extinction of the house light, a compound cue (auditory tone and stimulus light), and a 10-s (s) timeout during which no additional cocaine reinforcers can be delivered. Sessions ended when mice self-administered 60 infusions or at 120 min. Mice were considered to have acquired the cocaine-reinforced response when mice self-administered ≥15 reinforcers across three sessions (with ≥2:1 active/inactive lever press ratio) and a minimum of 10 total acquisition sessions. Drug intake (infusions), discrimination of the active versus inactive and time to reach criteria were measured.

After acquisition, mice were tested on an FR1 schedule with two consecutive sessions of descending unit doses of cocaine (1.0, 0.5, 0.25, 0.125, 0.063, and 0 mg/kg). This dose–response analysis measured the reinforcing properties of cocaine. Next, mice self-administered cocaine at the baseline 0.5 mg/kg unit dose for 2 days before progressive ratio testing. In order to measure motivation to take cocaine in the same mice, three counterbalanced unit doses (1.0, 0.5, and 0.25 mg/kg) were presented for two consecutive sessions under a progressive ratio schedule, wherein each successive infusion requires more lever press responses (1, 2, 4, 6, 9, 12, 16, 20…240). Sessions end after 4 or 1.5 h without acquiring a reinforcer. This test is used to measure motivation, which will be used to describe changes to the breakpoint ratio, or the last ratio obtained for a dose of cocaine. Mice were again returned to their baseline self-administration dose (0.5 mg/kg/infusion) for 2 days before extinction training. Here, all lever presses had no programmed consequences and mice were trained for 10 days or until ≤30% peak active responding at 0.5 mg/kg/infusion was reached. Following extinction, mice were tested for sensitivity to cue-induced reinstatement, a model of relapse where presentations of previously cocaine-associated cues invigorate responding on the previously cocaine-reinforced lever. Mice received one non-contingent cue presentation, followed by cues contingent on responding on the previously active lever.

### 2.5 Tissue collection

Mice exposed to adolescent CRD, as well as matching controls, were euthanized approximately 4 wks after the CRD or control paradigm ended by cervical dislocation and rapid decapitation at ZT6. Brains were flash frozen on dry ice and stored at −80°C until used for punching.

### 2.6 RNA-sequencing

The prefrontal cortex (PFC), nucleus accumbens (NAc), and suprachiasmatic nucleus (SCN) were punched and isolated from frozen brain tissue using a cryostat. Individual animals were each used as a sample (*n* = 4–8 per condition). Tissue was homogenized and total RNA was isolated using the RNeasy Plus Micro Kit (Qiagen). RNA quantity and quality were assessed using fluorometry (Qubit RNA High Sensitivity Assay Kit and Fluorometer; Invitrogen) and chromatography (Bioanalyzer and RNA 6000 Pico Kit; Agilent) respectively. Libraries were prepared with 10 ng of RNA from each sample using the Smartseq HT ultra-low input sample preparation kits (Illumina). Paired-end dual-indexed sequencing (75 bp) was performed using the NextSeq 500 platform (Illumina). A total of 30 million reads per sample was targeted. Sequencing was performed at the University of Pittsburgh Health Sciences Sequencing Core at UPMC Children’s Hospital of Pittsburgh.

FastQC v0.11.7 was used to assess the quality of the data. Per base sequence quality was high (Quality score generally >30) indicating good data quality. HISAT2 (HISAT2v2.1.0) was used to align reads to the reference (*Mus musculus* Ensembl GRCm38) using default parameters. The resulting bam files were converted to expression count data using HTSeq (HTSeq v0.10.0) with default union mode. Data normalization and gene filtering were performed separately for each region. Genes were retained for analysis if counts per million (CPM) was greater than 1 in 50% or more of the subjects within each group. All Y-chromosome genes were also eliminated from the analysis. A total of 10,386 (NAc), 10,220 (PFC), and 11,339 (SCN) annotated genes retained after filtering were used as the initial input dataset. The median of ratios normalization method in DESeq2 was then used to normalize for sequencing depth and RNA composition followed by a log2 transformation. Log2CPM values were used to calculate z-scores for expression for each transcript. Heatmaps were generated using z-scores to visualize changes in expression across group and brain region.

### 2.7 Statistical analyses

GraphPad Prism 10 was used. Data are expressed as mean ± SEM with *p* ≤ 0.05 considered significant and 0.05 < *p* ≤ 0.1 considered trending. Data were analyzed by two- or three-way ANOVAs for effects of CRD, sex and session/lever/dose and repeated measures (RM) were used when appropriate. ANOVAs with significant and trending interactions were followed by post-hoc tests with Bonferroni corrections for multiple comparisons. If a significant/trending interaction or effect of sex was found in a three-way RM ANOVA, data were separated by sex and two-way ANOVAs were performed in order to better examine the effect of CRD and the RM on the dependent variable. If no effect of sex (main or interaction) was found, data were collapsed for visualization and to increase power for subsequent analysis (ANOVA or unpaired two-tailed t-test), without sex as a factor. A Welch’s correction was used for unpaired two-tailed t-tests comparing groups that differed in variance, meaning that the F-test to compare variance between the two groups was significant. F-statistics from the higher order ANOVA (main effect of sex and interactions) are shown. Outliers over two standard deviations from the mean were excluded (*a priori*)*. F* or *t* < 1 is noted when appropriate for non-significant findings.

To examine gene expression changes by sex, at the approximate time of our behavioral assessments, a DE analysis was conducted by DESeq2 in each sex and brain region separately at ZT6. Genes were ranked for high DE based on q values and fold change in each brain region and sex. Genes were considered significantly DE with a q-value criteria of *q* < 0.05 to adjust for multiple comparisons. A fold change cut-off of 1.2 was used.

Metascape, a gene annotation and analysis resource, was used to identify enriched biological processes in the top DE genes (Zhou et al., 2019). To compare between groups, a significance threshold of *q* < 0.05 was used. *p* < 0.01 was used as a significance threshold to identify significantly enriched processes. For the background gene list in Metascape, we used a list of the 12,523 transcripts expressed in any of the 3 brain regions, which is more accurate than using the default full genome background. Further exploratory analysis was done on transcripts that did not pass the false discovery rate, instead using *p* < 0.05 to identify transcripts and identify enriched processes.

## 3 Results

### 3.1 Adolescent CRD increases exploratory drive and impairs spatial working memory

Mice were exposed to circadian rhythm disruption (CRD) during adolescence, which consisted of four 12 h shifts in the LD cycle occurring every 3 days, from P28-37 and behavior was tested in adulthood, ~4 weeks later. For most of the behaviors tested, analysis by two-way ANOVA revealed no interactions or effects of sex (*F*s < 1; latency to immobility: interaction *F*_(1,33)_ = 1.55, *p* = 0.22, sex *F*_(1,33)_ = 2.20, *p* = 0.15), so data were collapsed across sex and analyzed by unpaired two-tailed *t*-tests. For the LD box, where we did see a significant interaction, the data are separated by sex.

**Figure 1:**
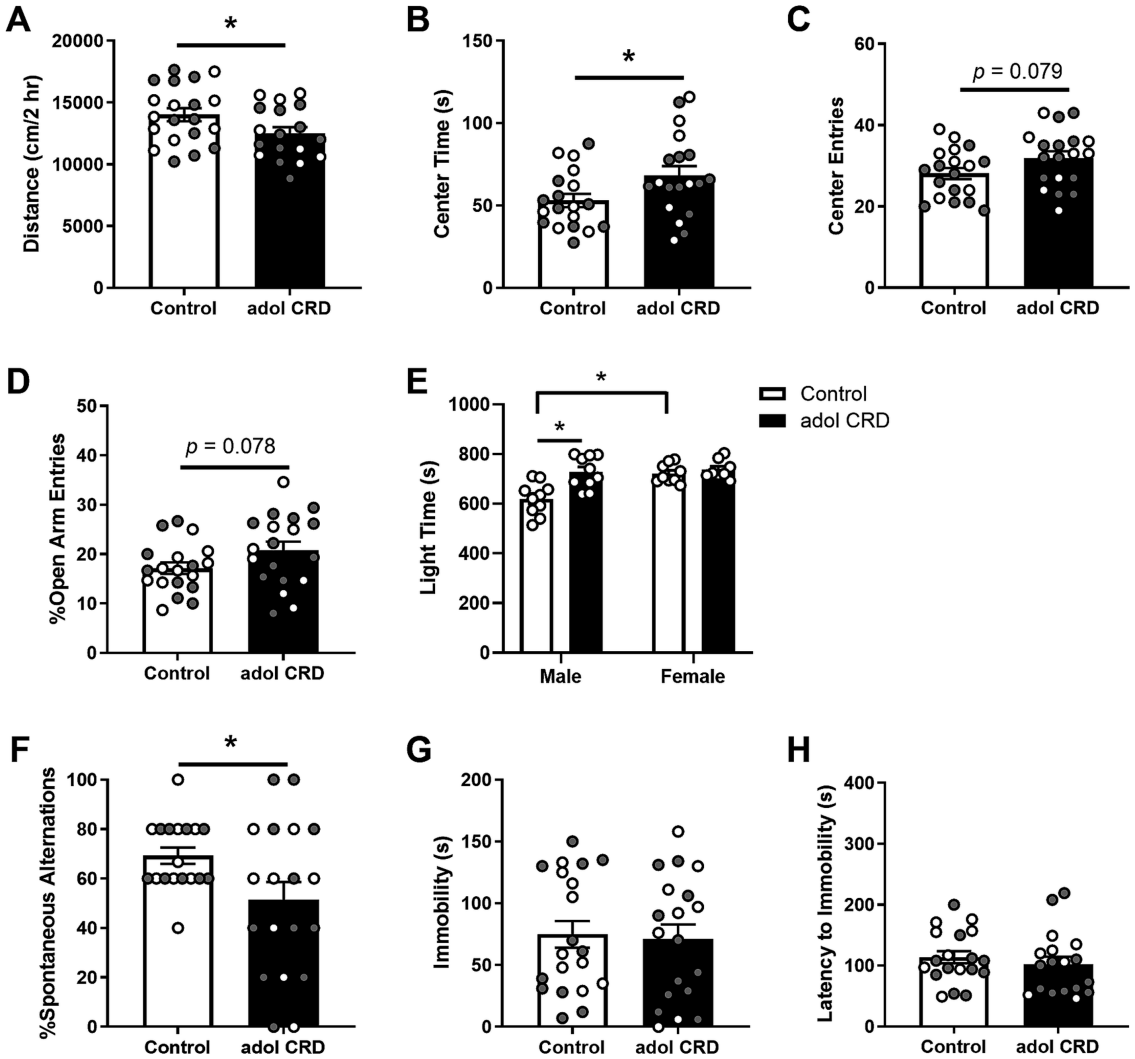
Adolescent CRD decreased locomotor activity in a novel environment **(A)**. Adolescent CRD tended to increase exploratory drive in the open field **(B,C)**, EPM **(D)**, and LD box **(E)**. Furthermore, adolescent CRD decreased spontaneous alternations in the T-maze, a measure of spatial working memory **(F)**. There was no effect of adolescent CRD in the FST **(G,H)**. Individual data points are shown for each sample with males indicated by gray-filled circles and females by open circles. Mean ± SEM, *n* = 18–20, *p ≤ 0.05.

We first determined if adolescent CRD affected locomotor activity in a novel environment and the exploratory drive of adult mice. Adolescent CRD decreased the distance traveled in a 2 h locomotor test in a novel environment (Figure 1A, *t*_(36)_ = 2.07, *p* = 0.046; Supplementary Figure 1), but increased exploratory drive in the open field, LD box, and EPM in adulthood (Figures 1B–E). Specifically, adolescent CRD increased the amount of time spent in the center of the open field (Figure 1B, *t*_(36)_ = 2.18, *p* = 0.036). There was also a trend for adolescent CRD to increase entries into the center of the open field (Figure 1C, *t*_(36)_ = 1.81, *p* = 0.079) and the percentage of open arm entries into the EPM (Figure 1D, *t*_(36)_ = 1.81, *p* = 0.078). Interestingly, unlike the other measures of exploratory drive, analysis by two-way ANOVA revealed an interaction between the effects of sex and adolescent CRD on time spent on the light side of the LD box (Figure 1E, *F*_(1, 33)_ = 6.44, *p* = 0.016). There was also an effect of sex (*F*_(1, 33)_ = 9.70, *p* = 0.004) and an overall effect of CRD (*F*_(1, 33)_ = 11.82, *p* = 0.002). Specifically for this measure in the LD box, adolescent CRD increased exploratory drive in adult males only. Also, there was a baseline sex difference, with female controls spending more time in the light relative to male controls, which may have caused a ceiling effect. Overall, circadian disruption during adolescence increased exploratory drive or risk-taking behaviors in adult mice, except for the LD box, where only males were affected.

To determine if adolescent CRD may affect cognitive function, we assessed the behavior of adult mice with a history of adolescent CRD in the T-maze. Adolescent CRD decreased spontaneous alternations in the T-maze suggesting that adolescent CRD impairs spatial working memory (Figure 1F, *t*_(25.37)_ = 2.27, *p* = 0.032).

To assess if adolescent CRD has long-lasting effects on helplessness-like behavior, we measured behavior in the FST and observed no differences in immobility (Figure 1G, *t* < 1) or latency to immobility (Figure 1H, *t* < 1) suggesting that adolescent CRD does not alter depressive-like behavior.

### 3.2 Adult CRD does not affect exploratory drive or spatial working memory

Next, we determined if CRD during adulthood, from P62-71, has similar effects when the same amount of time (~4 wks) was given between CRD and behavior testing. For most of the behaviors tested, analysis by two-way ANOVA revealed no interactions or effects of sex (*F*s < 1; locomotion: interaction *F*_(1,38)_ = 1.50, *p* = 0.23; center entries open field: sex *F*_(1,38)_ = 2.74, *p* = 0.11; open arm entries EPM: interaction *F*_(1,40)_ = 1.45, *p* = 0.24, sex *F*_(1,40)_ = 1.81, *p* = 0.19), so data were collapsed across sex and analyzed by unpaired two-tailed *t*-tests. For center time in the open field and latency to immobility for the FST, where we did see an effect of sex and interaction respectively, the data are separated by sex.

**Figure 2:**
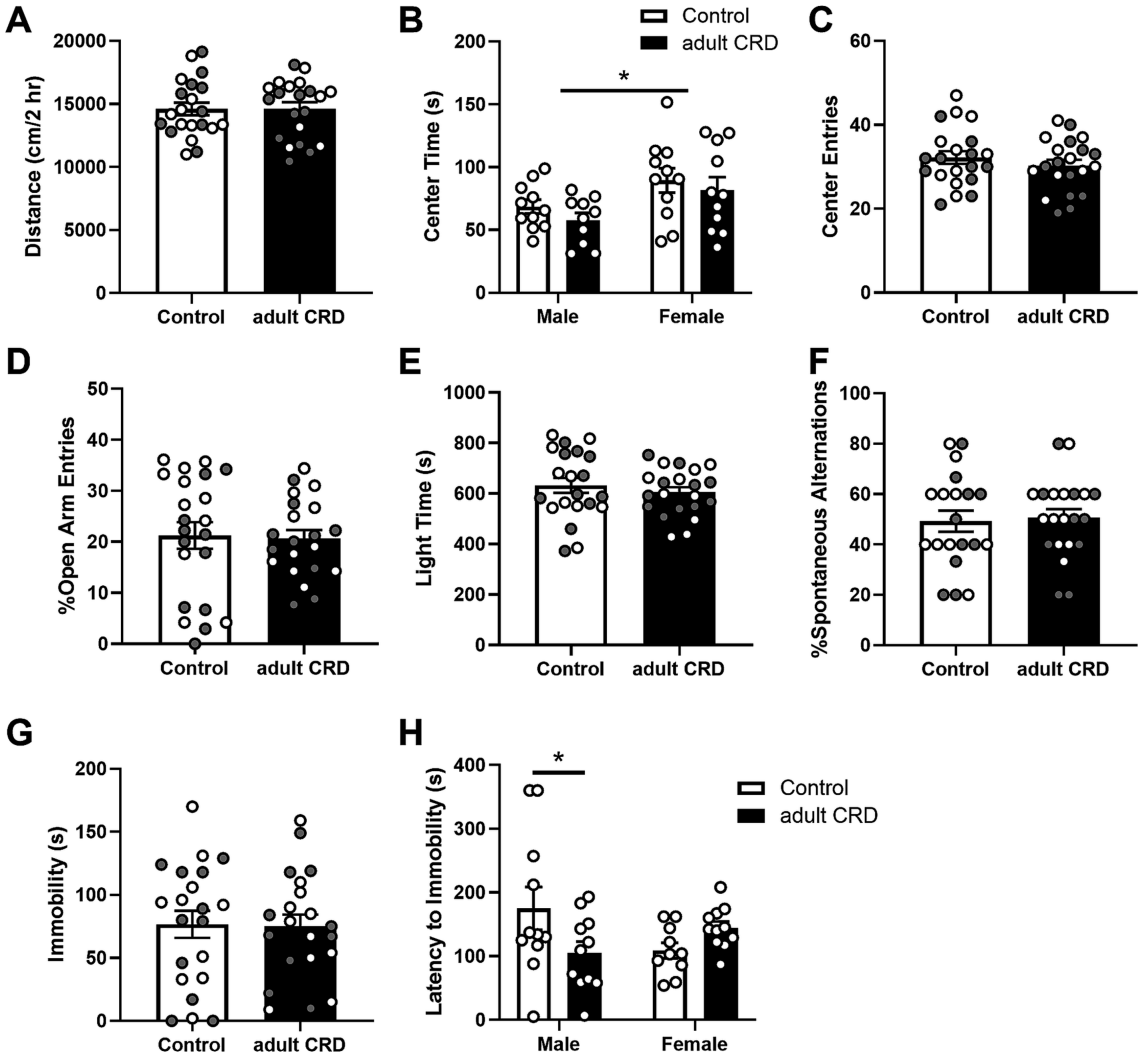
CRD during adulthood had no effect on locomotor activity in a novel environment **(A)**. Adult CRD had no effect on exploratory drive in the open field **(B,C)**, EPM **(D)**, or LD Box **(E)**. Furthermore, adult CRD had no effect on spontaneous alternations in the T-maze **(F)** or immobility in the FST **(G)**. However, adult CRD reduced latency to immobility in males in the FST **(H)**. Individual data points are shown for each sample with males indicated by gray-filled circles and females by open circles. Mean ± SEM, *n* = 20–22, *p ≤ 0.05.

Adult CRD had no effect on distance traveled in a 2 h locomotor test (Figure 2A, *t* < 1; Supplementary Figure 1). Analysis by two-way ANOVA revealed females overall spent more time in the center of the open field (Figure 2B, *F*_(1,39)_ = 7.56, *p* = 0.009). No effects of CRD (*F*_(1,39)_ = 1.27, *p* = 0.27) or interactions between sex and CRD (*F* < 1) were found for time spent in the center of the open field. Adult CRD also had no effect on the number of center entries in the open field (Figure 2C, *t* < 1). Furthermore, adult CRD had no effect on the percentage of open arm entries in the EPM (Figure 2D, *t* < 1) or time spent in the light side in the LD box test (Figure 2E, *t* < 1). Overall, circadian disruption in adulthood had no effect on locomotor activity or exploratory drive in adult mice that were tested ~4 wks after disruption occurred.

Adult CRD also had no effect on spatial working memory, with no change observed in the number of spontaneous alternations in the T-maze (Figure 2F, *t* < 1).

To assess if adult CRD has lasting effects on helplessness-like behavior, we assessed behavior in the FST. Adult CRD had no effect on immobility (Figure 2G, *t* < 1). Analysis by two-way ANOVA revealed an interaction between sex and adult CRD on latency to immobility (Figure 2H, *F*_(1, 39)_ = 6.35, *p* = 0.016). There were no overall effects of sex or adult CRD (*Fs* < 1). Bonferroni test showed a reduction in latency to immobility in adult CRD males, but not females.

### 3.3 Adolescent CRD, but not adult CRD increases cocaine reward

**Figure 3:**
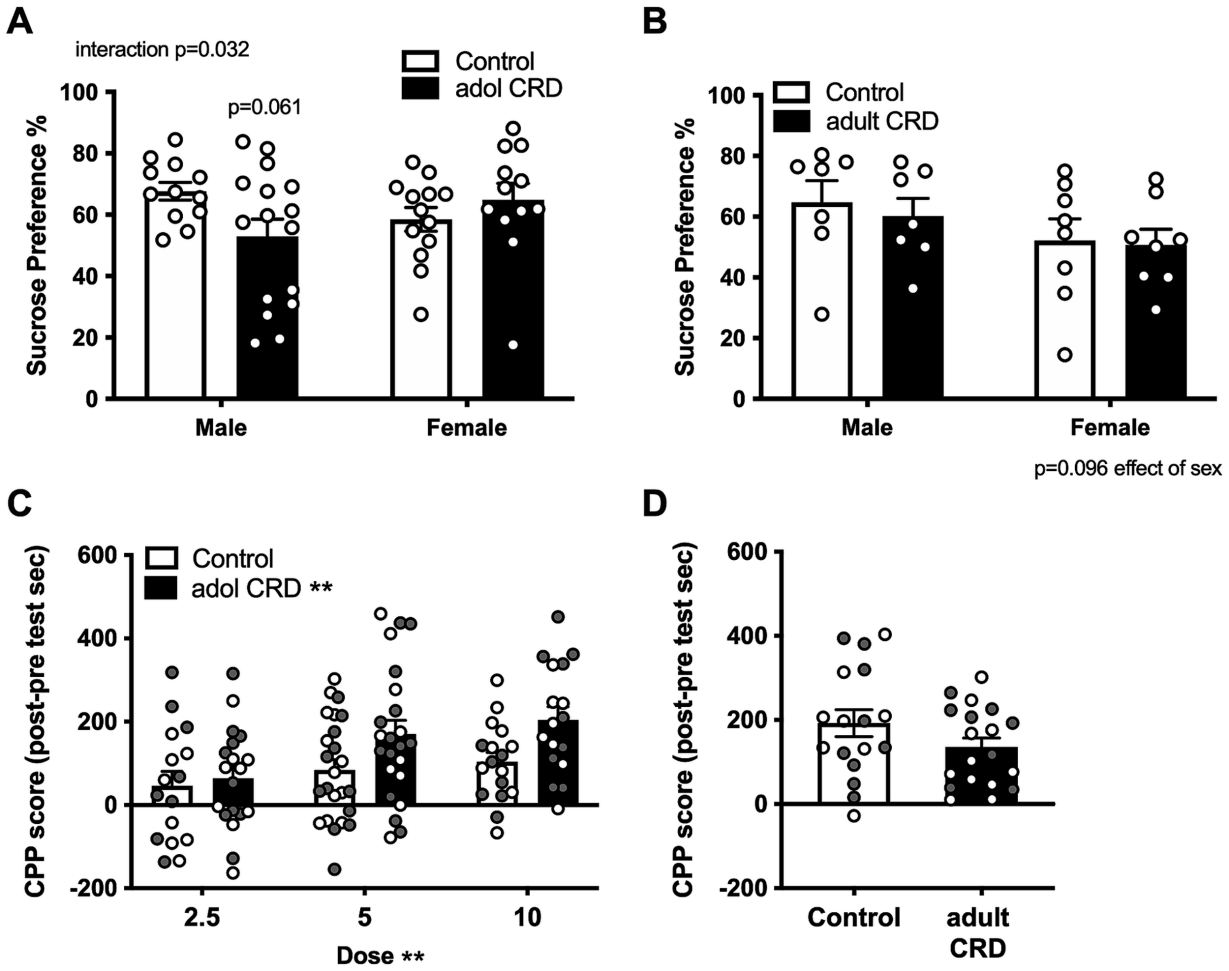
Effects of adolescent CRD and adult CRD on natural and cocaine reward. Adolescent CRD also altered sucrose preference, decreasing preference compared to controls in male mice, but not female mice **(A)**. There was no effect of adult CRD **(B)** on sucrose preference. Adolescent CRD **(C)**, but not adult CRD **(D)** increased cocaine CPP. Individual data points are shown for each sample with males indicated by gray-filled circles and females by open circles. Mean ± SEM, *n* = 12–16 for sucrose preference and 16–24 for CPP, *p ≤ 0.05.

Since increased exploratory drive or risk-taking-like behavior is associated with increased reward seeking, we then investigated if adolescent CRD specifically affects reward-related behaviors in mice. We first determined if adolescent CRD affects sucrose preference or natural reward. Here we found an interaction for the effects of sex and adolescent CRD on sucrose preference (Figure 3A, *F*_(1,49)_ = 4.87, *p* = 0.032). Post-hoc analyses revealed a trend for adolescent CRD to decrease sucrose preference in males, but adolescent CRD had no effect on sucrose preference in females. When adult mice experienced CRD and were tested ~4 wks later, sucrose preference was not altered relative to controls (Figure 3B). Analysis by two-way ANOVA revealed no effect of adult CRD or interaction (*F*s < 1), but there was a trend for decreased sucrose preference in all females relative to males (*F*_(1,26)_ = 2.99, *p* = 0.10). Overall, CRD during adolescence and adulthood seems to have a minimal effect on natural reward, with only a slight reduction in males following adolescent CRD.

We then investigated whether adolescent CRD and adult CRD may differentially affect drug reward by assessing cocaine CPP. Analysis by ANOVA revealed no interactions or effects of sex (*F*s < 1), so data were collapsed across sex and analyzed by a lower order ANOVA. Adolescent mice were conditioned to either 2.5, 5.0, or 10 mg/kg of cocaine. In a two-way ANOVA for effects of dose and adolescent CRD, as expected, higher cocaine doses resulted in greater CPP (Figure 3C, Effect of dose, *F*_(2, 110)_ = 5.35, *p* = 0.006). Interestingly, adolescent CRD overall increased cocaine CPP (*F*_(1, 110)_ = 7.76, *p* = 0.006). Adult mice were only conditioned to one dose of cocaine, the middle dose (5 mg/kg), since no behavioral effects had been seen thus far. CRD during adulthood had no effect on cocaine CPP at this dose (Figure 3D; *t*_(34)_ = 1.51, *p* = 0.14). Thus, adolescent CRD, but not adult CRD increased cocaine reward across sex.

### 3.4 Adolescent CRD has sex-specific effects on cocaine self-administration

Since differences in the sensitivity to cocaine reward after adolescent CRD may affect cocaine use, we then determined the effects of adolescent CRD on intravenous cocaine self-administration. For experiments assessing cocaine administration across RM (sessions, doses, or lever), data were analyzed by three-way ANOVAs for the effects of adolescent CRD, sex, and RM. If sex differences or an interaction with sex was observed, data were separated by sex and analyzed by two-way ANOVAs to better examine effects across our repeated measures (dose/session/lever).

Mice were trained to self-administer food before jugular catheters were placed to investigate whether operant conditioning is affected by adolescent CRD. A three-way ANOVA for the effects of adolescent CRD, sex, and session revealed no interactions or effect of sex (*F*s < 1), therefore sex was pooled. In a two-way ANOVA (adolescent CRD and session), all mice increased their responding for the reinforced lever as shown by an effect of session (Supplementary Figure 2, *F*_(4,376)_ = 190.1, *p* < 0.0001). We also found a trend for adolescent CRD to increase responding on the food-reinforced lever (*F*_(1,94)_ = 3.21, *p* = 0.077).

**Figure 4:**
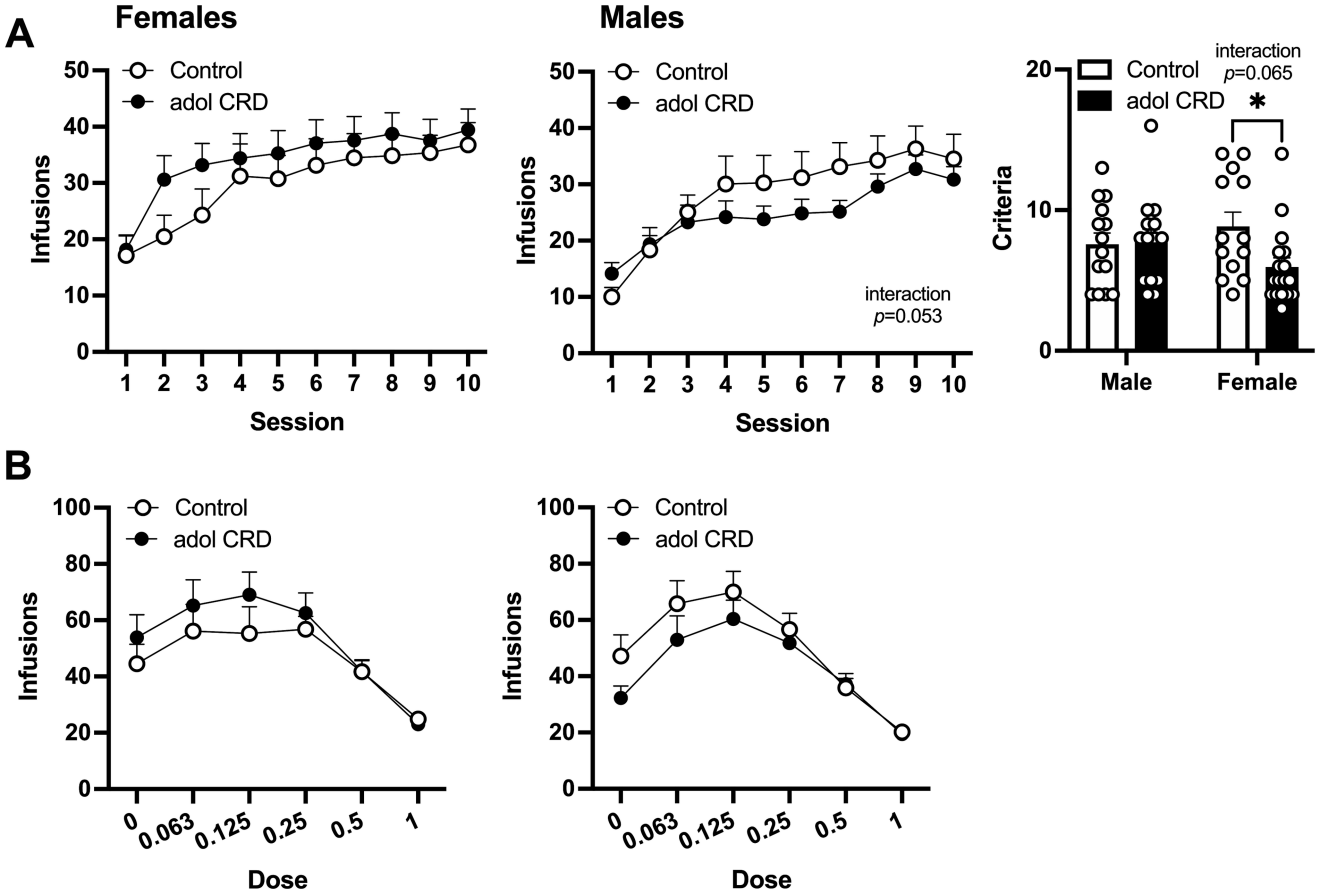
Effects of adolescent CRD on cocaine self-administration. **(A)** In females, adolescent CRD had no effect on the acquisition of cocaine self-administration (left). There was a trend for an interaction between the effects of adolescent CRD and session on the acquisition of cocaine self-administration in males (middle). Females with adolescent CRD reached the criteria for cocaine self-administration faster than controls (right). **(B)** Control and adolescent CRD female and male mice self-administered cocaine similarly in a dose–response curve. Mean ± SEM, *n* = 13–18, *p ≤ 0.05.

After mice recovered from jugular catheterization, they were trained to self-administer cocaine. In a three-way ANOVA, all mice acquired cocaine self-administration as shown by an increase in lever pressing across sessions (effect of session, *F*_(9,522)_ = 43.92, *p* < 0.0001). A trending effect of sex was also found (*F*_(1,58)_ = 2.78, *p* = 0.10), therefore two-way ANOVAs were performed for males and females separately. No change in cocaine self-administration was found in female adolescent CRD mice compared to controls (Figure 4A, effect of adolescent CRD *F* < 1). In males, there was a trend for an interaction between adolescent CRD and session (*F*_(9,261)_ = 1.90, *p* = 0.05). While there were no significant post-hoc effects of adolescent CRD on daily cocaine intake, this interaction suggests that male mice with a history of adolescent CRD acquire cocaine self-administration differently than controls. Interestingly, when sessions to reach criteria were quantified, a trend for an interaction was found (two-way ANOVA, adolescent CRD and sex, *F*_(1,54)_ = 3.56, *p* = 0.06). In post-hoc comparisons, female mice that were exposed to adolescent CRD reached criteria for self-administration significantly faster.

Following acquisition, a dose–response curve was used to measure the reinforcing properties of cocaine. Analysis by three-way ANOVA revealed an interaction between the effects of dose, sex and adolescent CRD (*F*_(5,285)_ = 2.38, *p* = 0.039). When analyzed separately, two-way ANOVAs did not reveal any significant effects of adolescent CRD within females (*F* < 1) or males (*F*_(1,29)_ = 1.05, *p* = 0.32) (Figure 4B). However, the three-way interaction above suggests that male and female mice have distinct responses to adolescent CRD in the context of a dose–response curve.

**Figure 5:**
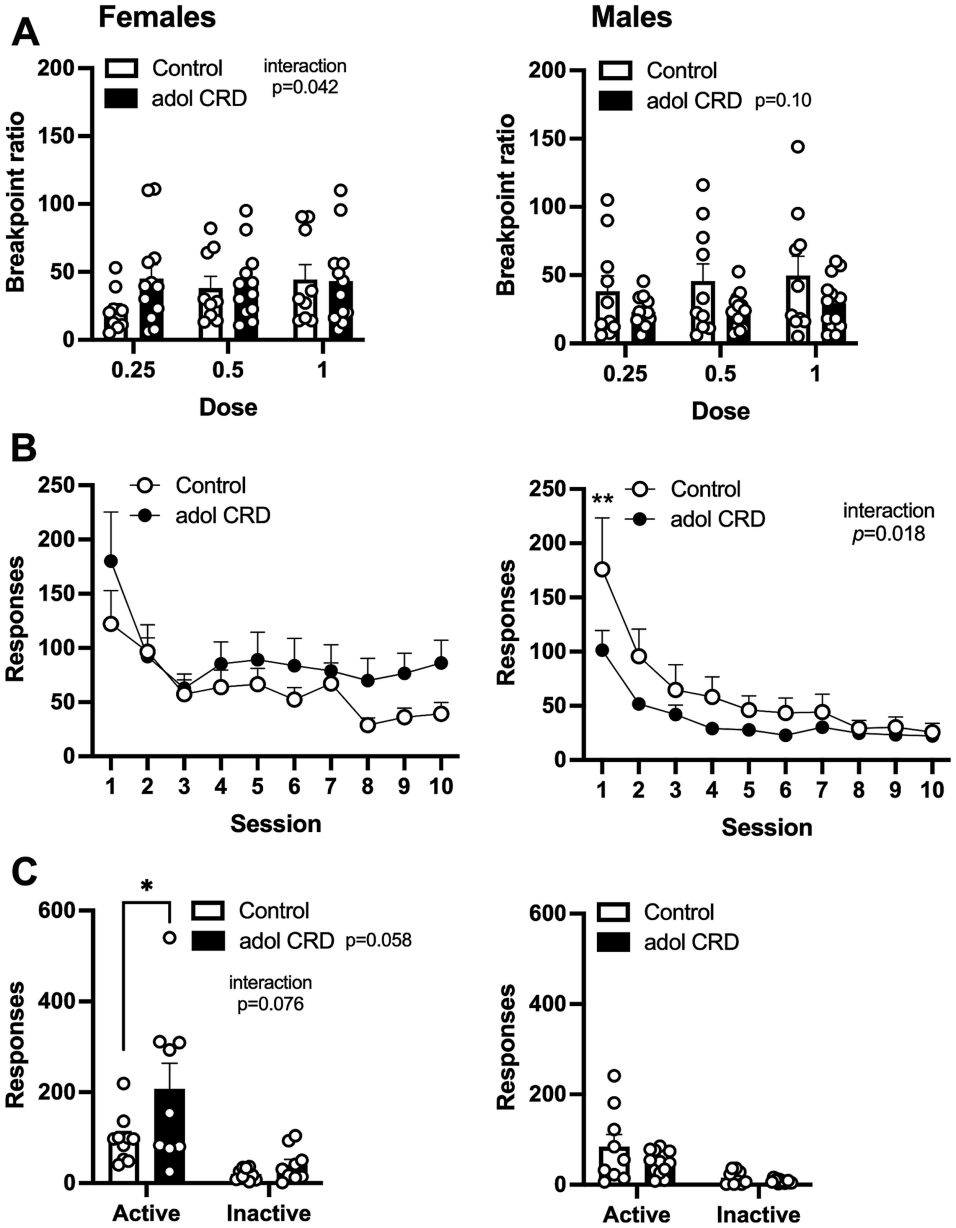
Effects of adolescent CRD on motivation, extinction, and cue-induced reinstatement. **(A)** There was a significant interaction between the effects of adolescent CRD and dose for motivation (breakpoint ratio) to self-administer cocaine in females, but no significant post-hocs were found (left). In males, there was a trend for adolescent CRD to reduce motivation to self-administer cocaine (right). **(B)** There was no effect of adolescent CRD on responding during extinction in female mice (left), while male mice with a history of CRD had reduced extinction responding (right). **(C)** For cue-induced reinstatement, responding was increased on the previously active lever in females with adolescent CRD (left), but no effect was found in males (right). Mean ± SEM, *n* = 9–13, *p ≤ 0.05.

Next, we assessed the effects of adolescent CRD on motivation for cocaine by determining the breakpoint ratio for responding during a progressive ratio schedule of reinforcement for three different doses of cocaine. A three-way ANOVA revealed a trending interaction between sex and adolescent CRD (*F*_(1,39)_ = 3.10, *p* = 0.09). In females, we found a significant interaction between the effects of cocaine dose and adolescent CRD (two-way ANOVA, Figure 5A, *F*_(2,38)_ = 3.44, *p* = 0.042), however no significant post-hoc comparisons were identified within any individual dose. For males, a trend was observed for an effect of adolescent CRD (*F*_(1,21)_ = 2.92, *p* = 0.10) with a decrease in breakpoint ratio, or motivation, in mice previously exposed to adolescent CRD.

We then assessed extinction and cue-induced reinstatement. In a three-way ANOVA, all mice extinguished responding as shown by a decrease in lever pressing across sessions when cocaine and cocaine-associated cues were withheld (Figure 5B, effect of session, *F*_(9,351)_ = 31.57, *p* < 0.0001). We also found a trending interaction between the effects of session, sex, and adolescent CRD (*F*_(9,351)_ = 1.79, *p* = 0.07). In females, adolescent CRD had no effect on extinction (two-way ANOVA, *F*_(1,19)_ = 1.15, *p* = 0.30), but in males, we observed an interaction between the effects of adolescent CRD and session (*F*_(9,180)_ = 2.30, *p* = 0.018). Males exposed to adolescent CRD responded less on the first session of extinction, compared to controls, indicating a possible decrease in frustrative non-reward or perseverative behavior on the previously active lever.

When we re-introduced the previously cocaine-associated cues, in the continued absence of intravenous cocaine, all mice exhibited cue-induced reinstatement by pressing the previously active lever more than the previously inactive lever (three-way ANOVA, main effect of lever, *F*_(1,37)_ = 49.65, *p* < 0.0001). Importantly, we also found a three-way interaction between lever, adolescent CRD and sex (Figure 5C, *F*_(1,37)_ = 5.38, *p* = 0.03). In subsequent two-way ANOVAs, there was an interaction between the effects of adolescent CRD and lever in females (*F*_(1,17)_ = 3.57, *p* = 0.08) as well as a trend for an overall effect of adolescent CRD (*F*_(1,17)_ = 4.12, *p* = 0.06). Female mice exposed to adolescent CRD showed an increase in cue-induced reinstatement, as shown by increased responding on the active lever, compared to controls. In males, there was no effect of adolescent CRD on cue-induced reinstatement (*F*_(1,20)_ = 2.50, *p* = 0.13).

Although some behavioral differences were small, and we identified many trending effects, these changes taken together suggest phenotypic changes after adolescent CRD. Overall, males with adolescent CRD tended to respond less for intravenous cocaine, with less motivation and reduced responding during extinction, while females with adolescent CRD tended to respond more, and faster, for intravenous cocaine and exhibited an increase in cue-induced reinstatement.

### 3.5 Adolescent CRD sex-dependently alters gene expression in the SCN

**Figure 6:**
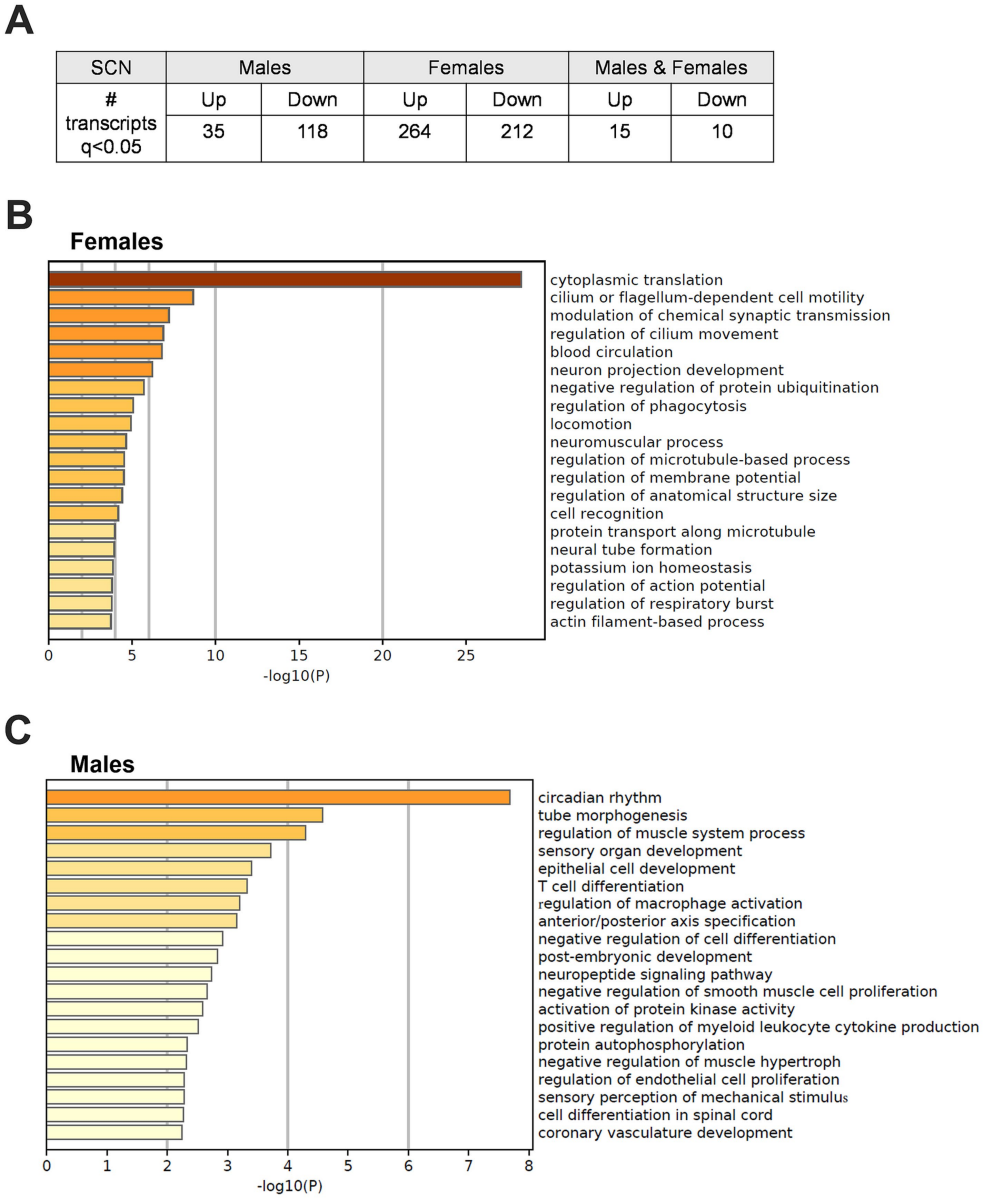
Differentially expressed transcripts following adolescent CRD in the SCN at ZT6. **(A)** Table listing the number of transcripts that are differentially expressed (DE) at ZT6 in adolescent CRD mice compared to controls in the SCN of males, females, or both combined at *q* < 0.05. **(B)** Metascape-derived heatmaps of biological processes enriched for DE transcripts (*q* < 0.05) in females and **(C)** males.

These studies have shown that adolescent CRD alters behavior related to risk-taking, reward, and cocaine self-administration. These adolescent CRD-induced behavioral changes could be caused by lasting changes in gene expression in circadian or reward-related brain regions. Given that most of our behavior assessments were performed in the early- to mid-light phase, we investigated changes in gene expression after adolescent CRD at around that same time of day (ZT6). We used a DE analysis to examine what transcripts were altered in female and male CRD mice. We found many more DE transcripts when females and males were examined separately instead of when they are pooled (Supplementary Table 1), suggesting distinct differences in these populations. Therefore, we did our analysis in each sex individually. In the SCN, 153 and 476 (*q* < 0.05) transcripts were DE in males and females, respectively (Figure 6A). When we examined these transcripts for enriched processes, we identified pathways in females involved in translation, microtubule-based movement, and membrane potential (Figure 6B). In males, we identified processes regulating circadian rhythms, intracellular signaling, immune responses and axon development as significantly enriched (Figure 6C). In the NAc and PFC at ZT6, transcripts were not DE at *q* < 0.05 in CRD mice relative to controls. We performed an exploratory analysis (Supplementary Figures 3–6) to identify potential DE transcripts in these regions at looser thresholds of *p* < 0.05 and *p* < 0.01 to investigate in future studies. See the Supplementary materials for a description of this exploratory analysis.

## 4 Discussion

The goal of our study was to investigate the effects of adolescent CRD on psychiatric-related behaviors and gene expression later in life. Mice exposed to 12 h shifts in the LD schedule during early adolescence (P28-37) exhibited increased exploratory drive and impaired working memory in adulthood. Mice as prey animals typically avoid open, elevated, or brightly lit areas of behavioral apparatuses, thus our findings suggest that adolescent CRD increases risk-taking behavior in mice. The persistent increase in risk-taking in adolescent CRD exposed mice relative to controls does not appear to be driven by differences in activity since disrupted mice moved less in a locomotor assay in a novel environment.

Previous studies in rodents indicate that the behavioral effects of environmentally induced CRD depend on the type of light cycle manipulation, its timing, and duration. In adult rodents, light cycle manipulations like constant light exposure or a fragmented LD schedule with more light overall, have increased exploratory drive/risk-taking behaviors in adult mice (Fonken et al., 2009; Richardson et al., 2021). Interestingly, the effects of exposing adult mice to fragmented LD schedules on risk-taking were not persistent and reverted after 2 weeks. Similarly, we did not observe persistent changes in risk-taking behaviors in adult mice tested ~4 wks after our CRD paradigm. Similar changes have been found when light cycle manipulations occur in juvenile mice. Disrupting circadian rhythms before weaning leads to increased exploratory drive and impaired working memory in adulthood (Borniger et al., 2014; Ameen et al., 2022). Some studies have found that LD cycle manipulations during adolescence alter exploratory drive in adult mice, but there have been few studies. For example, a chronic jet lag paradigm was shown to increase exploratory drive in the EPM, but this finding was specific to male Sandy mice, a line used to study aspects of schizophrenia (Cloutier et al., 2022). On the other hand, in female adolescent mice, exposure to light at night decreased exploratory drive in the open field in adulthood (Chen et al., 2021b). Here we found that 12 h shifts in the LD cycle during adolescence resulted in increased exploratory drive or risk-taking behavior in both sexes. The differences in the behavior findings in these studies may be due to differences in how phase shift paradigms and light at night during adolescence affect gene expression in the brain.

We also found that adolescent CRD impaired spatial working memory in adult mice while CRD in adulthood had no subsequent effect on spontaneous alternations in the T-maze. Interestingly, a recent study demonstrated that altering the photoperiod to model social jetlag in adolescent mice impaired novel object recognition and altered neural activity and gene expression in the hippocampus and amygdala (Bonilla et al., 2024). Similarly, exposing mice to a short-day LD cycle from gestation through P42 impaired memory and social behavior (Fang et al., 2021). Furthermore, LD cycle disturbances have been shown to alter cognitive behavior in adult rodents, but it is unclear how long impairments would last once CRD ends (Fernandez et al., 2014; Fuchs et al., 2023). Our results underscore the need for further research identifying the molecular links between CRD and different cognition behaviors across development.

Results from our group and others suggest that CRD at a critical period of development can have long-lasting effects on risky behavior and working memory in mouse models. However, more research is required to study the effects of environmental CRD on drug taking, especially for cocaine and opioids. While a few studies have shown that CRD affects drug intake, these studies have focused on alcohol and methamphetamine. For example, adolescent disruption escalates alcohol intake in adulthood, when animals have had prior exposure to alcohol (Gonzalez et al., 2023). CRD has also been shown to increase alcohol intake in adolescents (Gamsby et al., 2017) and increase methamphetamine consumption in adult rats pre-exposed to methamphetamine (Doyle et al., 2015). These studies confirm that CRD can significantly affect drug taking, but whether these effects transfer to other drugs of abuse and are exacerbated if disruption occurs before adulthood is not well understood.

We began to answer these questions here. In addition to studying exploratory behaviors, we measured cocaine reward and intake using cocaine CPP and intravenous cocaine self-administration, respectively. We found that adolescent CRD persistently increased cocaine preference in adulthood, and sex-specifically altered intravenous cocaine self-administration. Female adolescent CRD mice showed a greater propensity to self-administer cocaine, as well as increased motivation and cue-induced reinstatement. On the other hand, male adolescent CRD mice tended to self-administer less cocaine with reduced motivation and extinction responding. These results highlight the importance of studying volitional drug intake. While females showed a persistent increase in risky behavior across measures (exploratory drive, CPP and self-administration), males with a history of adolescent CRD showed a divergent behavioral response. Although unexpected, it is not unusual to find opposing results between conditioned place preference and self-administration (Larson et al., 2011) as in males here. These results suggest that although exploratory behaviors are increased in all mice with adolescent CRD, this unexpectedly only carries over to an increase in cocaine self-administration in female mice. This might be due to an anhedonic-like response developing in males after adolescent CRD where volitional, reward taking, such as in sucrose preference and cocaine self-administration, is reduced.

Further, it is possible that results were caused by stress due to the CRD rather than the CRD itself. However, other studies have demonstrated that stress during adolescence has different effects on behavior in adulthood. For example, chronic unpredictable stress during adolescence did not affect locomotor sensitization to amphetamine during adulthood (Kabbaj et al., 2002). Interestingly, other studies of sensitization following adolescence stress exposure reported similar findings for nicotine in males (McCormick and Ibrahim, 2007; Cruz et al., 2008). Stress during early adolescence has also been shown to increase anxiety-like behavior or reduce risk-taking-like behavior in adulthood (Albrecht et al., 2017). It is important to note that not everyone that experiences adolescent stress goes on to develop a substance use or other disorder in adulthood. Therefore, it is essential to continue investigating different forms of adverse experiences during development to determine their specific impact.

To identify possible mechanisms by which CRD during adolescence affects behaviors later in life, we measured gene expression in brain regions relevant to circadian rhythms, mood, and reward, including the SCN, PFC, and NAc. Since we found sex-specific effects on drug-related behavior during the early/mid light phase, it is possible that changes at that TOD (ZT6) are driving behavioral changes. Therefore, we measured DE between control and adolescent CRD mice at ZT6 in male and female mice and found that the transcripts affected by adolescent CRD were largely distinct across sex and brain region. The SCN was particularly affected, with more DE transcripts identified at *q* < 0.05. Thus, our observed behavioral changes may be mediated by the SCN or its effects on downstream regions. Interestingly, the identified transcripts altered by CRD were non-overlapping between males and females, suggesting sex-dependent mechanisms. For example, synaptic transmission and microtubule/actin processes are enriched after CRD in females, while immune responses are especially prevalent in males. These differences could then cause further differences in downstream brain regions, including cortical or midbrain regions, possibly occurring via connections in the paraventricular nucleus (PVT).

One possible mechanism for changes downstream in the extra-SCN brain are modulations in hormone signaling, which is also a prevalent method for the SCN to synchronize rhythms in the brain and body. We found a general upregulation in processes related to peptide and hormone secretion and transport in the SCN, suggesting possible changes in important hormones, such as arginine vasopressin and corticosterone, that might affect downstream brain regions. It is important to note that downstream brain regions could also be responding to adolescent CRD independently from the SCN since the retina also transmits light information through the perihabenular nucleus of the thalamus (Fernandez et al., 2018). Further studies are needed to better understand how changes in the SCN after adolescent CRD are affecting the rest of the brain, especially the PFC and NAc.

Downstream of the SCN we observed minor changes in the NAc and PFC that did not survive false discovery rate correction. Our exploratory analysis (Supplementary material) found several interesting results, but future studies with greater sample sizes and more power will be needed to confirm the relevance of these findings. At very minimal statistical cut-offs (*p* < 0.05), we found that in the PFC, transcripts with altered gene expression are associated with the extracellular matrix and kinase signaling in both males and females. These parallel changes may emphasize how critical neural activity and structure in the PFC is during adolescence when significant structural and synaptic maturation are occuring (Bourgeois et al., 1994; Huttenlocher and Dabholkar, 1997; Gourley et al., 2012; Koss et al., 2014). We also found many sex-specific changes, which could help explain the sex differences we found in reward-related behavior. These are discussed further in the Supplementary material.

Taken together, the transcripts and enriched processes that were DE at ZT6 provide insight into how adolescent CRD might alter the SCN. We hypothesize that the SCN is directly impacted by light cycle changes and through changes in neuronal activity or neuropeptide/hormone signaling, communicates those changes to reward-related brain regions, such as the PFC and NAc. However, it is important to note that since the changes found in the PFC and NAc did not reach significance thresholds, the SCN is likely predominately affecting other brain regions, or is affecting many regions, including the PFC and NAc in parallel. Small scale changes across many cortical or midbrain regions could act in concert to change behavior.

In future studies, it will also be critical to investigate how behavior and gene expression change across the 24 h day. Diurnal rhythms in mood- and reward-related behavior are extremely common, for example motivation and drug intake peak during the dark or active phase in nocturnal rodents (Baird and Gauvin, 2000; Abarca et al., 2002; Roberts et al., 2002; Sleipness et al., 2005). Future studies might identify more pronounced behavioral effects after adolescent CRD if behavior is measured at other times.

In addition, while the CRD paradigm used here serves as a valuable model for how multiple, large disruptions to circadian rhythms during adolescence can have differential, long-lasting effects relative to adults, these 12 h LD shifts are not an environmental disruption commonly experienced by humans. Adolescents and adults experience large circadian/sleep disruptions due to school, work, and social obligations for years, not weeks and abnormal LD schedules are typically caused by indoor lighting at night and the use of electronics. In the future, more translationally relevant light paradigms could be used, such as constant light, fragmented light, or social jet lag paradigms, wherein animals are exposed to different schedules during the weekdays and weekends.

Overall, these experiments found that LD cycle disruptions persistently altered reward-related behavior, but only when disruption occurred during adolescence. Furthermore, females showed increased exploratory drive and reward-related behavior, while males showed increases in exploratory drive and cocaine preference, but reductions in volitional reward intake, such as sucrose preference and cocaine self-administration. This was the first study, to our knowledge, to measure cocaine self-administration in mice after environmental CRD. Intravenous self-administration is a highly translational behavioral model of substance use in mice and provides the best evidence of changes in propensity to abuse substances, drug taking and drug seeking in animal models. We also identified changes in gene expression after adolescent CRD in the SCN. These results emphasize how important and long-lasting the effects of CRD during adolescence are. Experiments like these are needed to identify novel intervention strategies to alleviate disruption during adolescence and new molecular targets for drug development.

## Data Availability

Raw and processed RNA-seq data presented in this study are deposited into the NCBI GEO database (accession no. GSE282275).
