# Supplementary material for: Adolescent circadian rhythm disruption increases reward and risk-taking
Source: Front Neurosci. 2024 Dec 16;18:1478508. doi: 10.3389/fnins.2024.1478508 (PMC11683121; doi:10.3389/fnins.2024.1478508)
Supplement: Supplementary file 1 [file Data_Sheet_1.docx]

**Supplemental Materials**

**Exploratory RNA-seq results that did not survive false discovery rate correction**

To further explore sequencing results, particularly in the NAc and PFC where transcripts were not significantly altered after CRD under a FDR, additional exploratory analysis was used. *p* < 0.05 was used to identify transcripts and identify enriched processes. Volcano plots were used to visualize all DE transcripts at ZT6 in each brain region and sex. Volcano plots were used to choose 3 outlying DE transcripts for further investigation based on a *p*-value cutoff and/or high fold change.

In the NAc and PFC at ZT6, transcripts were not DE at q < 0.05 in CRD mice relative to controls. We performed an exploratory analysis (**Supplementary Figures 3-6**) to identify potential DE transcripts in these regions, as well as the SCN, at looser thresholds of *p* < 0.05 and *p* < 0.01 to investigate in future studies.

We found DE transcripts at ZT6: 1251, 411, and 93 (*p* < 0.05) in males in the SCN, PFC, and NAc respectively and 2058, 277 and 116 in females (**Supplemental Figure 3A**). Venn diagrams show all DE transcripts for each region within females (**Supplemental Figure 3B**) and males (**Supplemental Figure 3C)**. Similar patterns in overlap (not shown) exist in transcripts that were specifically up or downregulated in adolescent CRD mice.

The most overlapping DE transcripts in males and females after adolescent CRD were found in the SCN (186 transcripts in the SCN, 13 PFC, 3 NAc), indicating that the effects of adolescent CRD are more sex dependent in the PFC and NAc. As expected, this overlap carried over to enriched biological processes, where reported processes are only shared across sex in the SCN, but not the NAc and PFC (**Supplementary Figures 4-5**). The shared processes enriched in the SCN of males and females are largely upregulated and tend to regulate synapse function, as well as peptide and hormone secretion and transport (**Supplementary Figure 4**). Due to the number of groups investigated, we will only discuss trends in a few highly relevant, overall enriched processes.

In the SCN of females, we tended to see overall enriched processes related to synaptic neurotransmission. Synapse function (organization, structure, assembly), trans-synaptic signaling (neurotransmission, plasticity), translation and biosynthesis at the synapse, and microtubule-based movement (extracellular transport, cytoskeletal organization, intracellular transport) were all enriched. In the PFC of female mice with a history of adolescent CRD, processes involved in IL-8 production, DNA repair, axonogenesis and plasma membrane fusion/cell adhesion were enriched at ZT6 (**Supplementary Figure 5**). In the NAc, very few processes were identified (**Supplementary Figure 5**), but ion transport, calcium-mediated signaling, and catecholamine metabolism were enriched in females with adolescent CRD.

In males, in the SCN we found overall enriched processes in synapse function (organization, structure, assembly), axon development, intracellular signaling, and learning and memory. In the PFC of male mice with adolescent CRD, processes were enriched for axonogenesis, GPCR (G-protein coupled receptor) signaling, tight junction assembly and organization, Wnt signaling, and trans-synaptic signaling. In males in the NAc, DE transcripts were enriched for processes that regulate DNA recombination and protein oligomerization.

In addition to enriched processes, we selected 3 transcripts that were highly upregulated and highly downregulated after adolescent CRD in the SCN, PFC and NAc. Volcano plots were used to identify transcripts of interest (shown in green, **Supplementary Figure 6**) for each brain region and sex. Heatmaps of z-scores in expression were used to visualize differences in expression in control and CRD animals **(Supplementary Figure 3)**. Overall, in the SCN, DE genes in male and female mice with adolescent CRD encode ion channels and neuropeptides, as well as GTPase activating proteins, which are found only in males. In the PFC, females had DE transcripts that encode extracellular matrix proteins and proteins that regulate kinase activity, while DE transcripts in male adolescent CRD mice include ribosomal transcripts and peptides. Lastly, in the NAc of female CRD mice, DE genes were mostly involved with DNA structure and function, while DE genes in males were ion channels and genes that regulate immune responses.

In our exploratory analysis, we observed many changes in the NAc and PFC (DE p < 0.05), which could contribute to altered behavior following adolescent CRD. In the PFC, transcripts with altered gene expression are associated with the extracellular matrix and kinase signaling in both males and females in the PFC. These parallel changes emphasize how critical neural activity and structure in the PFC is during adolescence. Adolescence is a critical period defined by significant structural and synaptic maturation, including proliferation, pruning and stabilization of synapses and dendritic spines in the PFC (Bourgeois et al., 1994; Huttenlocher and Dabholkar, 1997; Gourley et al., 2012; Koss et al., 2014). It is believed that this reorganization opens a window of vulnerability to insults during adolescence, especially for the development of psychiatric disorders. Our exploratory findings suggest that adolescent CRD may alter structural remodeling in the PFC, which has long-term effects on PFC activity and signaling and in turn contributes to altered reward-related behavior later in life.

In the PFC and NAc, we also found more sex-specific changes in gene expression after adolescent CRD at ZT6 than in the SCN. This could help explain the sex differences we found in reward-related behavior, since there was almost no overlap in DE transcripts and enriched processes. More specifically, many enriched processes are identified in both the NAc and PFC that regulate signaling and activity, but these are not found in every sex and region. In females, gene expression changes in the PFC after adolescent CRD suggest alterations in kinase signaling, immune responses and extracellular matrix functions, while in the NAc females have enriched processes that are specific to signaling mechanisms (calcium signaling, ion transport, catecholamine metabolism). These changes could be contributing to increased reward and drug taking in females with adolescent CRD. In male mice, changes in gene expression after adolescent CRD similarly suggest altered immune responses, kinase signaling and synapse organization (dendrite and dendritic spine development). However, DE transcripts in males also specifically regulate glutamatergic, GABAergic, GPCR, Wnt and trans-synaptic signaling after adolescent CRD in the PFC, but not the NAc. These results could explain why males show more exploratory drive or risk-taking behavior in concert with a more anhedonic-like phenotype, with less sucrose consumption and less cocaine-taking behaviors, since anhedonia is often found in mood disorders, which are highly relevant to the PFC (Heshmati and Russo, 2015; Hare and Duman, 2020; Otsuka et al., 2020; Codeluppi et al., 2023).

**References**

Bourgeois, J.-P., Goldman-Rakic, P. S., and Rakic, P. (1994). Synaptogenesis in the Prefrontal Cortex of Rhesus Monkeys. *Cereb. Cortex* 4, 78–96. doi: 10.1093/cercor/4.1.78

Codeluppi, S. A., Xu, M., Bansal, Y., Lepack, A. E., Duric, V., Chow, M., et al. (2023). Prefrontal cortex astroglia modulate anhedonia-like behavior. *Mol. Psychiatry* 28, 4632–4641. doi: 10.1038/s41380-023-02246-1

Gourley, S. L., Olevska, A., Warren, M. S., Taylor, J. R., and Koleske, A. J. (2012). Arg Kinase Regulates Prefrontal Dendritic Spine Refinement and Cocaine-Induced Plasticity. *J. Neurosci.* 32, 2314–2323. doi: 10.1523/JNEUROSCI.2730-11.2012

Hare, B. D., and Duman, R. S. (2020). Prefrontal cortex circuits in depression and anxiety: contribution of discrete neuronal populations and target regions. *Mol. Psychiatry* 25, 2742–2758. doi: 10.1038/s41380-020-0685-9

Heshmati, M., and Russo, S. J. (2015). Anhedonia and the Brain Reward Circuitry in Depression. *Curr. Behav. Neurosci. Rep.* 2, 146–153. doi: 10.1007/s40473-015-0044-3

Huttenlocher, P. R., and Dabholkar, A. S. (1997). Regional differences in synaptogenesis in human cerebral cortex. *J. Comp. Neurol.* 387, 167–178. doi: 10.1002/(SICI)1096-9861(19971020)387:2<167::AID-CNE1>3.0.CO;2-Z

Koss, W. A., Belden, C. E., Hristov, A. D., and Juraska, J. M. (2014). Dendritic remodeling in the adolescent medial prefrontal cortex and the basolateral amygdala of male and female rats. *Synapse* 68, 61–72. doi: 10.1002/syn.21716

Otsuka, T., Thi Le, H., Kohsaka, A., Sato, F., Ihara, H., Nakao, T., et al. (2020). Adverse Effects of Circadian Disorganization on Mood and Molecular Rhythms in the Prefrontal Cortex of Mice. *Neuroscience* 432, 44–54. doi: 10.1016/j.neuroscience.2020.02.013

**Supplementary Figures**


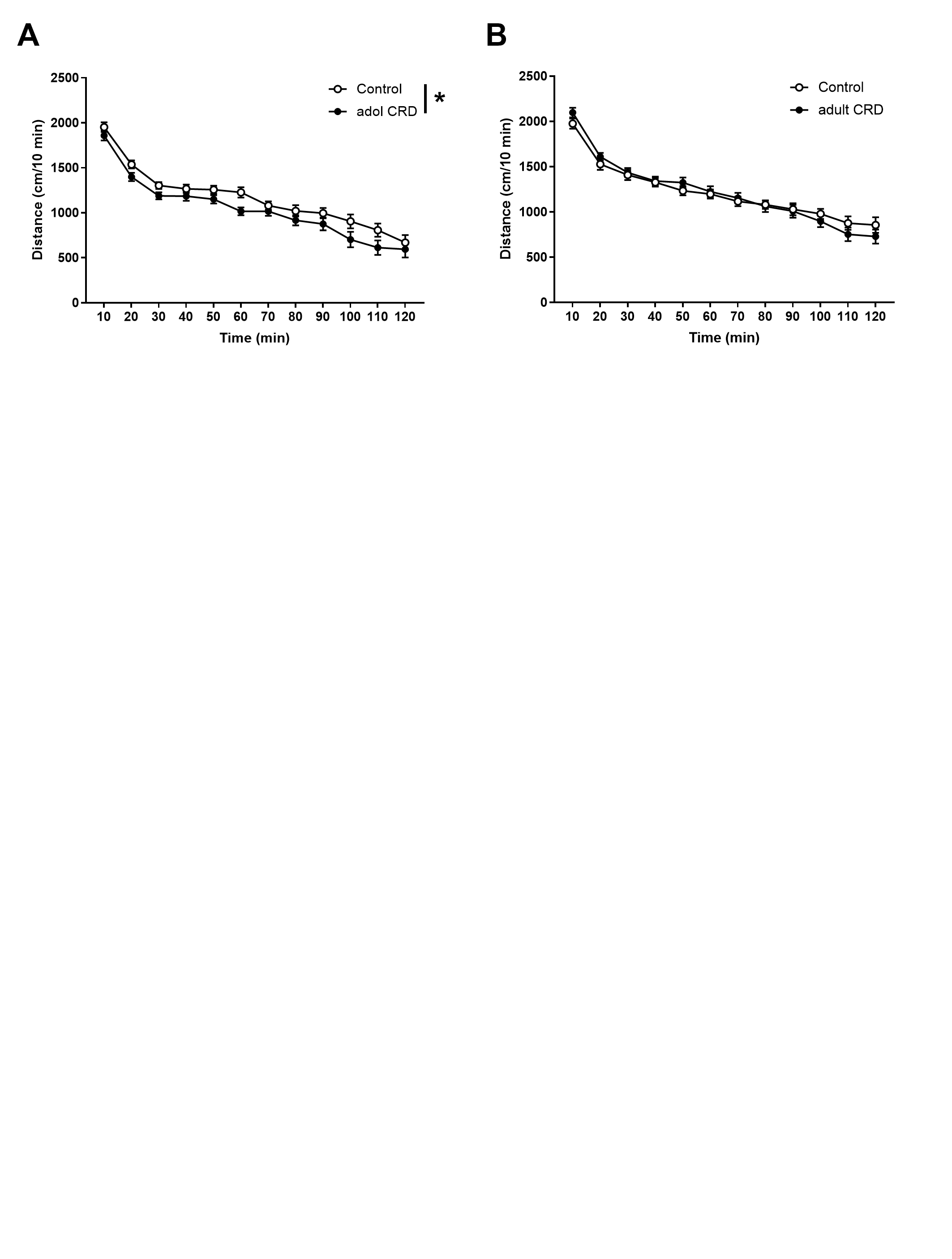


**Supplementary Figure 1**. Locomotor response to novelty across 10 min bins during a 2-hour test in adolescent CRD **(A)** and adult CRD animals **(B)**. We found a main effect of adolescent CRD but no interaction (main effect of adolescent CRD, *F*_(1, 36)_ = 4.28, *p* = 0.046, interaction time x adolescent CRD *F*_(11, 396)_ = 0.66, *p* = 0.78). We found no effect of adult CRD and a trend for an interaction (main effect of adult CRD, *F*_(1, 40)_ = 0.0011, *p* = 0.97, interaction time x adult CRD *F*_(11, 440)_ = 1.63, *p* = 0.089). Bonferroni post-hoc tests comparing control and adult CRD mice at each time bin were not significant. Mean ± SEM, n = 20-22.


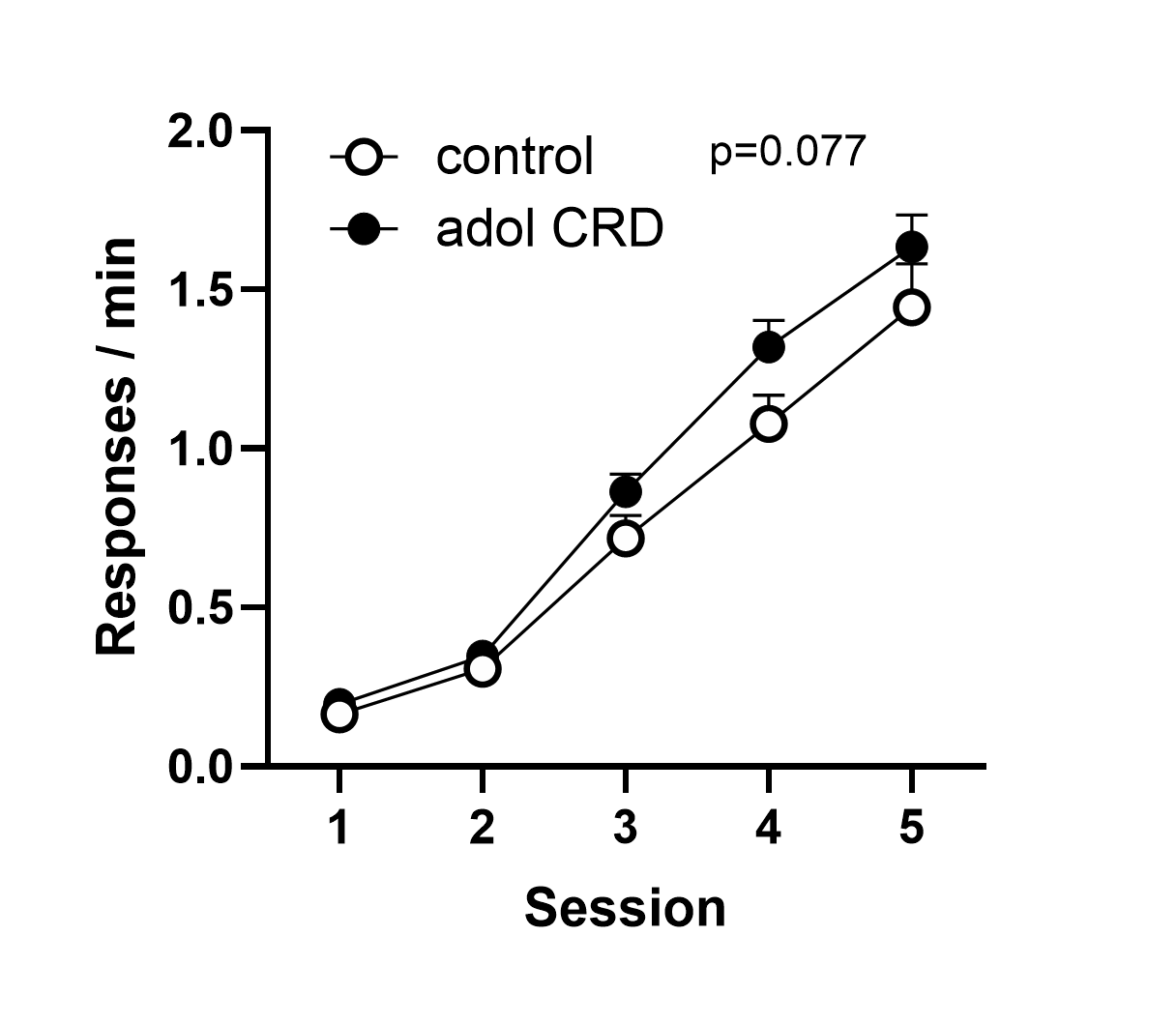


**Supplementary Figure 2**. Food self-administration prior to jugular catheterization. There was a trend for adolescent CRD to increase responding on the food reinforced lever.


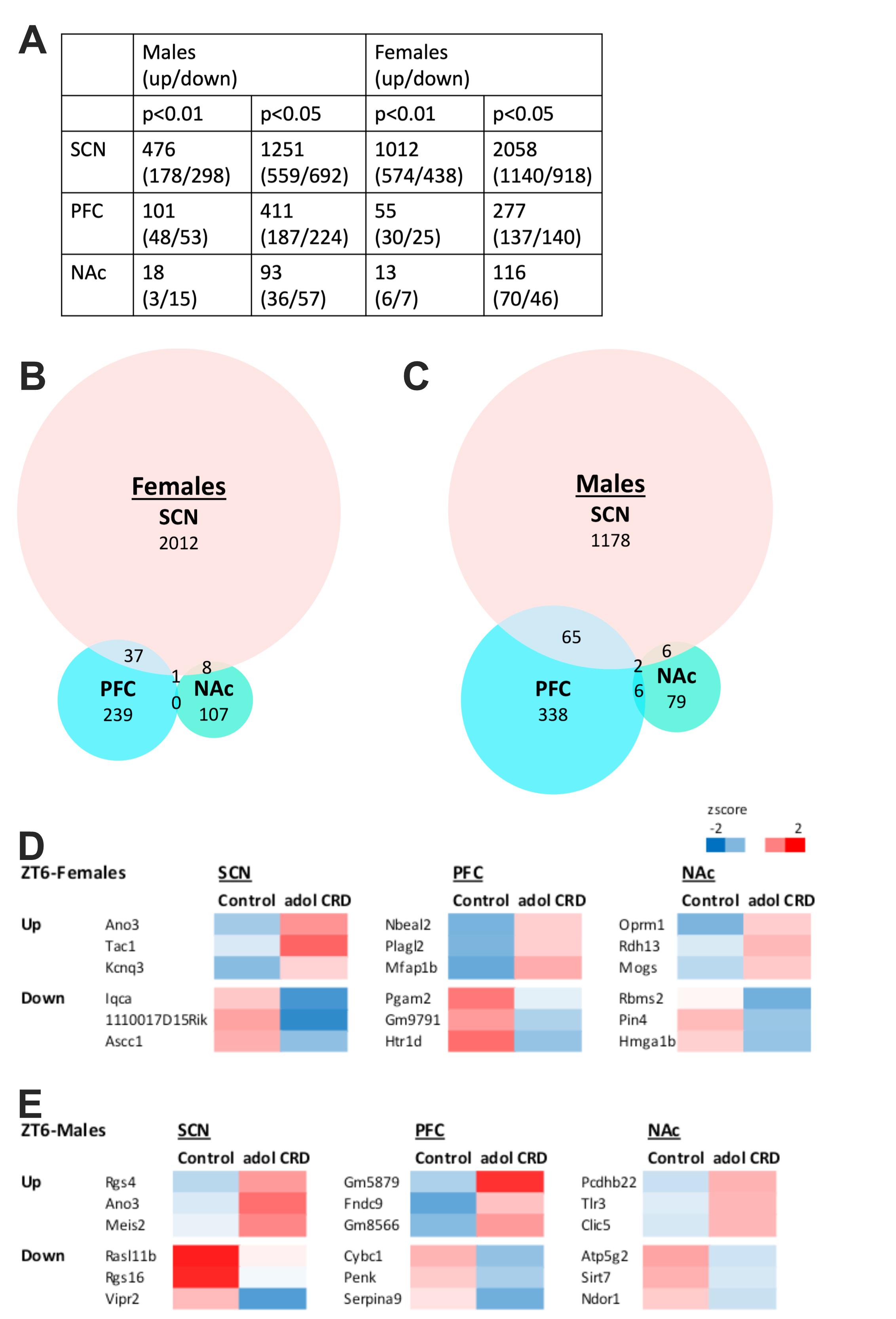

**Supplementary Figure 3**. Differentially expressed transcripts in adolescent CRD mice vary depending on brain region and sex at ZT6. Table listing the number of transcripts that are differentially expressed (DE) at ZT6 in adolescent CRD mice compared to controls in the SCN, PFC and NAc. DE transcripts at ZT6 are shown, with up and downregulated transcripts listed underneath in brackets **(A)**. Venn diagrams show how DE transcripts (*p* < 0.05) overlap across brain region in females **(B)** and males **(C)**. Heat maps of z-scores show patterns of gene expression in control and adolescent CRD mice for 3 of the most up and downregulated transcripts at ZT6 in females **(D)** and males **(E)**. Transcripts were chosen as outlying transcripts in volcano plots (Supplementary Figure 5).


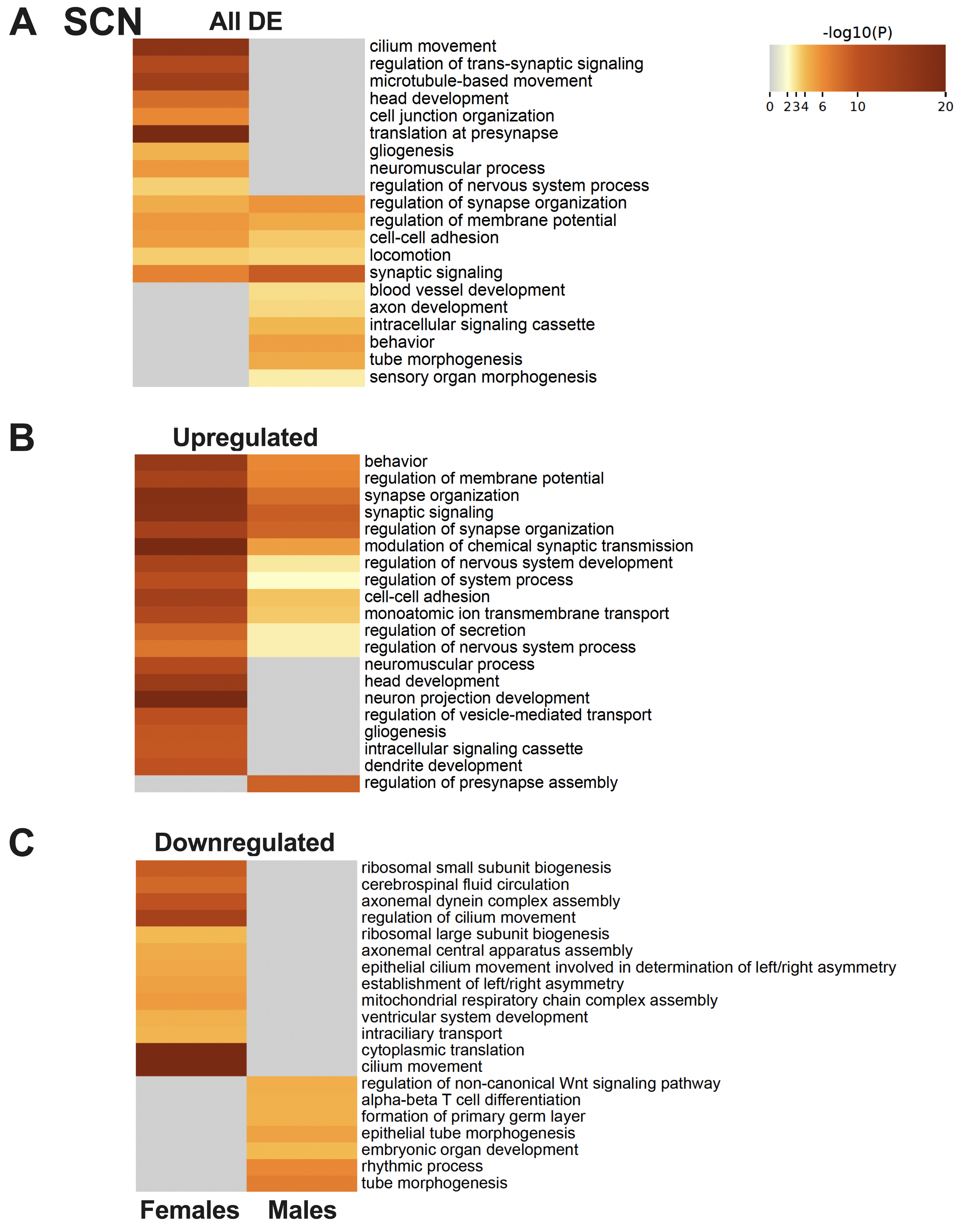


**Supplementary Figure 4.** The top 20 enriched biological processes are shown for all differentially expressed transcripts in the SCN after adolescent CRD **(A).** Enriched biological processes are also shown for transcripts that are upregulated **(B)** and downregulated **(C)** in mice with adolescent CRD compared to controls in the SCN at ZT6. Throughout, enriched processes are shown for male and female mice, with overlap when appropriate, and the significance of these processes is shown as a heatmap for –log10(p-value).


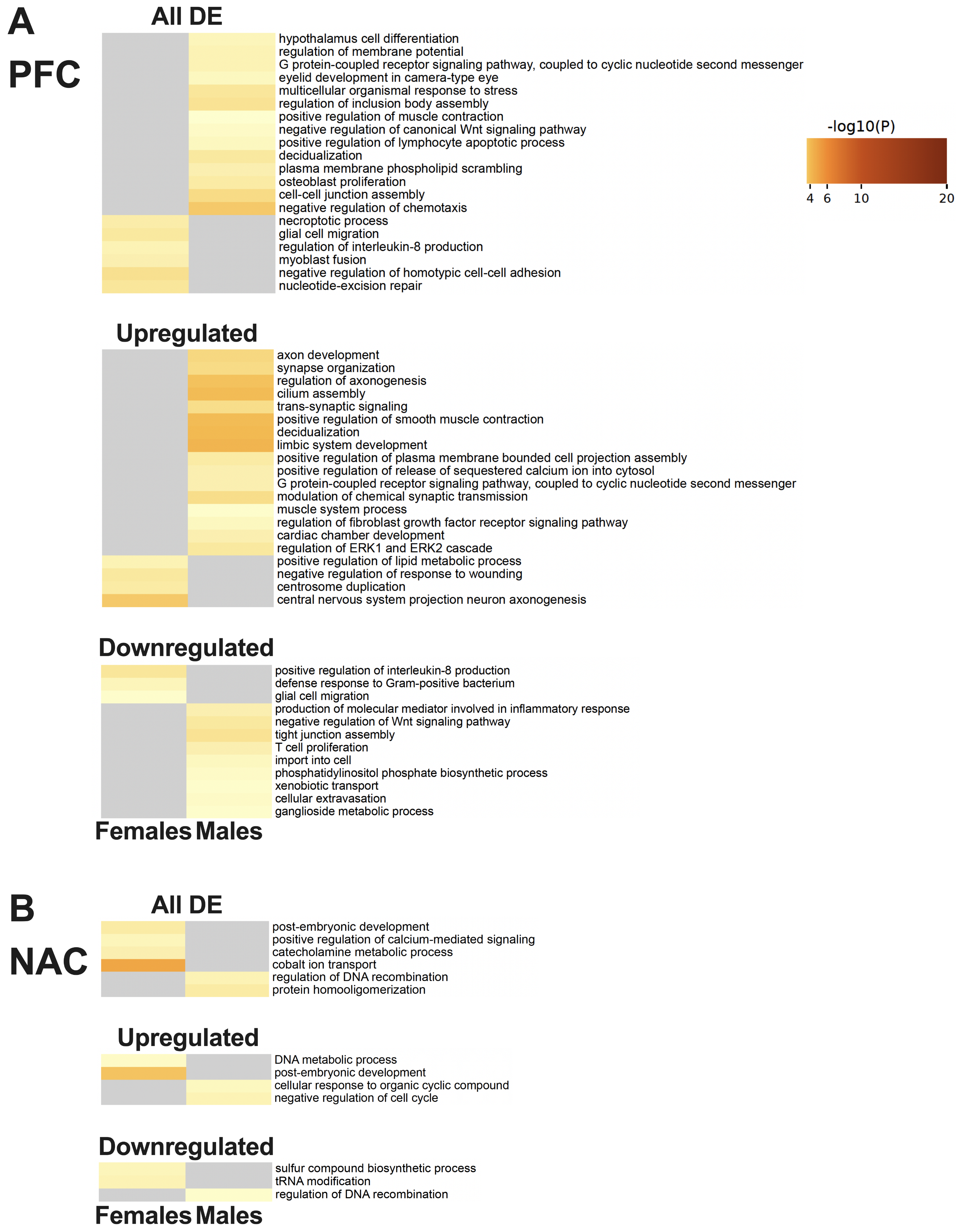


**Supplementary Figure 5.** The top enriched biological processes are shown for differentially expressed (p<0.05) (top), upregulated (middle) and downregulated (bottom) transcripts in the PFC **(A)** and NAc **(B)** after adolescent CRD at ZT6. Throughout, enriched processes are shown for male and female mice with significance indicated by a heatmap for –log10(p-value).


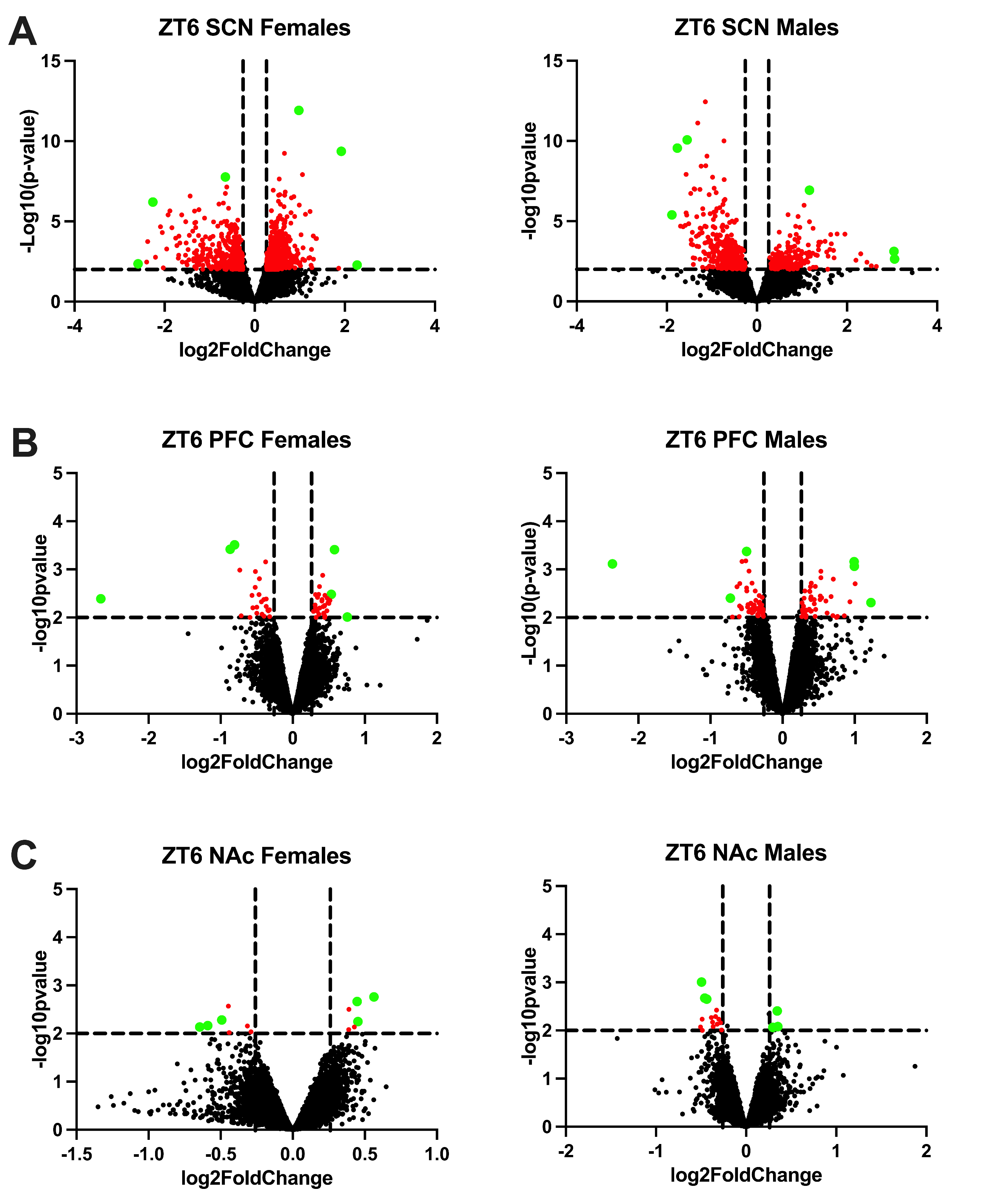


**Supplementary Figure 6.** Differentially expressed transcripts between control and adolescent CRD at ZT6 is shown in volcano plots for the SCN **(A)**, PFC **(B)**, and NAc **(C)** of female (left) and male mice (right). Horizontal dashed lines represent a cutoff of p < 0.01 and vertical dashed lines represent log2FC cutoffs of ≤ -0.26 or ≥ 0.26. Transcripts meeting both criteria are represented by red circles, with highly differentially expressed transcripts shown in green.

**Supplementary Tables**

**Supplementary Table 1:** List of significantly up and down regulated transcripts at different thresholds in males, females, or both combined.

|  | Males  (up/down) | | | Females  (up/down) | | | Males and Females  (up/down) | | |
| --- | --- | --- | --- | --- | --- | --- | --- | --- | --- |
|  | p<0.01 | p<0.05 | q<0.05 | p<0.01 | p<0.05 | q<0.05 | p<0.01 | p<0.05 | q<0.05 |
| SCN | 476  (178/298) | 1251  (559/692) | 153  (35/118) | 1012  (574/438) | 2058  (1140/918) | 476  (264/212) | 389  (207/182) | 899  (429/470) | 25  (15/10) |
| PFC | 101  (48/53) | 411  (187/224) | 0 | 55  (30/25) | 277  (137/140) | 0 | 17  (11/6) | 113  (76/37) | 0 |
| NAc | 18  (3/15) | 93  (36/57) | 0 | 13  (6/7) | 116  (70/46) | 0 | 5  (4/1) | 18  (11/7) | 0 |

**Supplementary Table 2**: Tables showing sample sizes for each group. Does not include behavioral measures where individual data points are shown in the figures. Sample sizes can decrease over the course of intravenous self-administration, due to the presence of occasional outliers and attrition of catheter patency.

|  | Female control | Female shift | Male control | Male shift |
| --- | --- | --- | --- | --- |
| I.V. : Acquisition | 13 | 18 | 16 | 15 (1) |
| I.V. : Dose response | 13 | 17 | 15 (1) | 16 |
| I.V. : Extinction | 10 | 11 | 9 | 13 |
| RNAseq | 4 | 5 | 5 | 5 |

**Supplementary Table 3-7:** Tables showing statistical analysis, test statistic, p-value, mean difference and confidence intervals for each figure 1-5.

| Figure | Test | Test statistic | p-value | Mean difference ± SE | 95% CI |
| --- | --- | --- | --- | --- | --- |
| Figure 1A | Unpaired t-test | T (36) = 2.070 | **0.0457** | -1509 ± 729.2 | -2988 to -30.34 |
| Figure 1B | Unpaired t-test | T (36) = 2.177 | **0.0361** | 15.16 ± 6.963 | 1.035 to 29.28 |
| Figure 1C | Unpaired t-test | T (36) = 1.809 | 0.0788 | 3.842 ± 2.124 | -0.4654 to 8.150 |
| Figure 1D | Unpaired t-test | T (36) = 1.812 | 0.0783 | 3.682 ± 2.032 | -0.4384 to 7.803 |
| Figure 1E | 2-way ANOVA | F (1, 33) = 9.701 | **0.0038 – Main effect of sex** | -55.70 ± 17.88 | -92.08 to -19.32 |
|  |  | F (1, 33) = 11.82 | **0.0016 – Main effect of CRD** | -61.48 ± 17.88 | -97.86 to -25.10 |
|  |  | F (1, 33) = 6.443 | **0.0160 – Interaction** | -90.78 | -163.5 to -18.02 |
| Figure 1F | Unpaired t-test | T (25.37) = 2.270 | **0.0319** | -17.68 ± 7.932 | -33.78 to -1.578 |
| Figure 1G | Unpaired t-test | T (37) = 0.2249 | 0.8233 | -3.534 ± 15.71 | -35.37 to 28.31 |
| Figure 1H | Unpaired t-test | T (35) =0.7324 | 0.4688 | -11.45 ± 15.64 | -43.20 to 20.29 |

**Supplementary Table 4:**

| Figure | Test | Test statistic | p-value | Mean difference ± SE | 95% CI |
| --- | --- | --- | --- | --- | --- |
| Figure 2A | Unpaired t-test | T (40) =0.03392 | 0.9731 | 24.19 ± 713.1 | -1417 to 1465 |
| Figure 2B | 2-way ANOVA | F (1, 39) = 7.561 | **0.0090- Main effect of sex** | -22.56 ± 8.203 | -39.15 to -5.964 |
|  |  | F (1, 39) = 1.266 | 0.2675 – Main effect of CRD | 9.228 ± 8.203 | -7.363 to 25.82 |
|  |  | F (1, 39) = 0.03999 | 0.8425 - Interaction | 3.281 | -29.90 to 36.46 |
| Figure 2C | Unpaired t-test | T (40) =0.9301 | 0.3579 | -1.905 ± 2.048 | -6.044 to 2.234 |
| Figure 2D | Unpaired t-test | T (35.59) =0.1958 | 0.8459 | -0.6025 ± 3.109 | -6.882 to 5.677 |
| Figure 2E | Unpaired t- test | T (40) =0.7610 | 0.4511 | -27.41 ± 36.03 | -100.2 to 45.40 |
| Figure 2F | Unpaired t-test | T (39) =0.2555 | 0.7997 | 1.385 ± 5.421 | -9.579 to 12.35 |
| Figure 2G | Unpaired t- test | T (40) =0.09772 | 0.9226 | -1.381 ± 14.13 | -29.94 to 27.18 |
| Figure 2H | 2-way ANOVA | F (1, 39) = 0.4187 | 0.5214 – Main effect of sex | 13.50 ± 20.87 | -28.71 to 55.72 |
|  |  | F (1, 39) = 0.6526 | 0.4241 – Main effect of CRD | 16.86 ± 20.87 | -25.35 to 59.07 |
|  |  | F (1, 39) = 6.352 | **0.0159 - Interaction** | 105.2 | 20.77 to 189.6 |

**Supplementary Table 5:**

| Figure | Test | Test statistic | p-value | Mean difference ± SE | 95% CI |
| --- | --- | --- | --- | --- | --- |
| Figure 3A | 2-way ANOVA | F (1, 49) = 0.08173 | 0.7762 – Sex | -1.367 ± 4.781 | -10.97 to 8.241 |
|  |  | F (1, 49) = 0.7573 | 0.3884 – CRD | 4.161 ± 4.781 | -5.447 to 13.77 |
|  |  | F (1, 49) = 4.873 | **0.0320 – Interaction** | 21.11 | 1.891 to 40.32 |
| Figure 3B | 2-way ANOVA | F (1, 26) = 2.993 | **0.0955 – Sex** | 11.05 ± 6.389 | -2.079 to 24.19 |
|  |  | F (1, 26) = 0.2095 | 0.6510 – CRD | 2.924 ± 6.389 | -10.21 to 16.06 |
|  |  | F (1, 26) = 0.06072 | 0.8073 – Interaction | 3.149 | -23.12 to 29.42 |
| Figure 3C | 2-way ANOVA | F (2, 110) = 5.352 | **0.0061 – Dose** |  |  |
|  |  | F (1, 110) = 7.759 | **0.0063 – CRD** | -68.46 ± 24.58 | -117.2 to -19.75 |
|  |  | F (2, 110) = 1.016 | 0.3653 – Interaction |  |  |
| Figure 3D | Unpaired t-test | T (34) = 1.506 | 0.1412 | -56.90 ± 37.77 | -133.7 to 19.86 |

**Supplementary Table 6:**

| Figure | Test | Test statistic | p-value | Mean difference ± SE | 95% CI |
| --- | --- | --- | --- | --- | --- |
| Figure 4A - Females | 2-way ANOVA | F (9, 261) = 19.64 | <0.0001 – Main effect of session |  |  |
|  |  | F (1, 29) = 0.7037 | 0.4084 – Main effect of CRD | -4.331 ± 5.163 | -14.89 to 6.228 |
|  |  | F (9, 261) = 1.028 | 0.4179 – Interaction |  |  |
| Figure 4A - Males | 2-way ANOVA | F (9, 261) = 25.17 | <0.0001 – Main effect of session |  |  |
|  |  | F (1, 29) = 0.7508 | 0.3933 – Main effect of CRD | 3.518 ± 4.060 | -4.786 to 11.82 |
|  |  | F (9, 261) = 1.898 | **0.0526 - Interaction** |  |  |
| Figure 4A - Criteria | 2-way ANOVA | F (1, 54) = 0.1186 | 0.7318 – Main effect of sex | 0.2849 ± 0.8271 | -1.373 to 1.943 |
|  |  | F (1, 54) = 2.646 | 0.1097 – Main effect of CRD | 1.345 ± 0.8271 | -0.3130 to 3.004 |
|  |  | F (1, 54) = 3.555 | 0.0647 - Interaction | -3.119 | -6.436 to 0.1973 |
| Figure 4B - Females | 2-way ANOVA | F (5, 140) = 25.59 | <0.0001 – Main effect of session |  |  |
|  |  | F (1, 28) = 0.5290 | 0.4731 – Main effect of CRD | -6.017 ± 8.272 | -22.96 to 10.93 |
|  |  | F (5, 140) = 1.034 | 0.4003 – Interaction |  |  |
| Figure 4B – Males | 2-way ANOVA | F (5, 145) = 43.10 | <0.0001 – Main effect of session |  |  |
|  |  | F (1, 29) = 1.045 | 0.3150 – Main effect of CRD | 6.948 ± 6.796 | -6.950 to 20.85 |
|  |  | F (5, 145) = 1.633 | 0.1550 - Interaction |  |  |

**Supplementary Table 7:**

| Figure | Test | Test statistic | p-value | Mean difference ± SE | 95% CI |
| --- | --- | --- | --- | --- | --- |
| Figure 5A – Females | 2-way ANOVA | F (2, 38) = 2.216 | 0.1230 – Main effect of session |  |  |
|  |  | F (1, 19) = 0.5484 | 0.4680 – Main effect of CRD | -8.532 ± 11.52 | -32.65 to 15.58 |
|  |  | F (2, 38) = 3.441 | **0.0423 – Interaction** |  |  |
| Figure 5A – Males | 2-way ANOVA | F (2, 42) = 2.840 | 0.0697 – Main effect of session |  |  |
|  |  | F (1, 21) = 2.916 | 0.1024 – Main effect of CRD | 19.08 ± 11.17 | -4.154 to 42.31 |
|  |  | F (2, 42) = 0.4526 | 0.6390 – Interaction |  |  |
| Figure 5B - Females | 2-way ANOVA | F (9, 171) = 11.21 | <0.0001 – Main effect of session |  |  |
|  |  | F (1, 19) = 1.151 | 0.2969 – Main effect of CRD | -27.43 ± 25.57 | -80.94 to 26.09 |
|  |  | F (9, 171) = 1.292 | 0.2443 – Interaction |  |  |
| Figure 5B – Males | 2-way ANOVA | F (9, 180) = 23.56 | <0.0001 – Main effect of session |  |  |
|  |  | F (1, 20) = 2.608 | 0.1220 – Main effect of CRD | 23.87 ± 14.78 | -6.962 to 54.70 |
|  |  | F (9, 180) = 2.295 | **0.0184 – Interaction** |  |  |
| Figure 5C - Females | 2-way ANOVA | F (1, 17) = 26.10 | <0.0001 – Main effect of lever | 122.4 ± 23.96 | 71.85 to 172.9 |
|  |  | F (1, 17) = 4.116 | 0.0584 – Main effect of CRD | -65.50 ± 32.29 | -133.6 to 2.616 |
|  |  | F (1, 17) = 3.572 | 0.0759 – Interaction | -90.56 | -191.6 to 10.53 |
| Figure 5C – Males | 2-way ANOVA | F (1, 20) = 25.52 | <0.0001 – Main effect of lever | 55.34 ± 10.95 | 32.49 to 78.19 |
|  |  | F (1, 20) = 2.499 | 0.1296 – Main effect of CRD | 21.28 ± 13.46 | -6.800 to 49.36 |
|  |  | F (1, 20) = 1.458 | 0.2414 – Interaction | 26.45 | -19.25 to 72.15 |
